# MacAma: Multi‐AI Agent as a Co‐Scientist for Automated Meta‐Analysis

**DOI:** 10.1002/smmd.70045

**Published:** 2026-07-29

**Authors:** Yilin Yuan, Pingping Li, Yang Wang, Boyuan Zheng, Yingshuang Liu, Dongjin Yang, Hai Lin, Min Wu, Qi Zhao, Jianwei Shuai, Gen Yang

**Affiliations:** ^1^ State Key Laboratory of Nuclear Physics and Technology School of Physics Peking University Beijing China; ^2^ Joint Medical Engineering Interdisciplinary Research Center Wenzhou Institute UCAS, and the Second Affiliated Hospital of Wenzhou Medical University Wenzhou Zhejiang China; ^3^ Zhejiang Key Laboratory of Soft Matter Biomedical Materials Wenzhou Institute University of Chinese Academy of Sciences Wenzhou Zhejiang China; ^4^ Beijing National Laboratory for Condensed Matter Physics, Institute of Physics Chinese Academy of Sciences Beijing China; ^5^ School of Physical Sciences University of Chinese Academy of Sciences Beijing China; ^6^ Wenzhou Key Laboratory of Biophysics Wenzhou Institute University of Chinese Academy of Sciences Wenzhou Zhejiang China; ^7^ Department of Medicine Harvard Medical School and Brigham and Women’s Hospital Boston Massachusetts USA; ^8^ Tianfu Jincheng Laboratory Chengdu China; ^9^ School of Computer Science and Software Engineering University of Science and Technology Liaoning Anshan China; ^10^ School of Public Health Wenzhou Medical University Wenzhou China; ^11^ The University of Hong Kong‐Shenzhen Hospital Shenzhen China

**Keywords:** large language models, literature screening, meta‐analysis, prompt engineering, research automation

## Abstract

Meta‐analysis is fundamental to evidence‐based medicine, yet traditional workflows remain labor‐intensive and susceptible to bias. Although LLM‐based research agents offer opportunities for workflow automation, they often lack the data fidelity and methodological traceability required for rigorous quantitative evidence synthesis, particularly when parsing multimodal scientific charts. To address this challenge, we introduce MacAma, a semi‐automated multi‐agent framework for protocol‐constrained and human‐verifiable meta‐analysis. MacAma operationalizes selected PRISMA 2020 reporting items, PICOS‐based eligibility logic, and SYRCLE risk‐of‐bias domains as structured prompts, decision rules, output fields, and audit records. Critically, MacAma adopts a risk‐aware automation strategy: Lower risk, repetitive, and protocol‐driven tasks, such as literature screening and drafting, are delegated to AI agents, whereas high‐impact steps that directly affect effect‐size estimation and statistical conclusions, such as quantitative chart‐data extraction, remain subject to expert verification. *In a preclinical radiotherapy case study evaluating tumor‐related immune outcomes and metastatic potential mediated by circulating tumor cells, MacAma achieved competitive screening performance in the evaluated benchmark and reduced the manual screening burden by over 80% within the current workflow. The case study further demonstrates how structured agent outputs, predefined criteria, and audit records can* *support transparent screening, data extraction, statistical synthesis, and manuscript drafting*. These results suggest that MacAma may provide a scalable and auditable framework for AI‐assisted meta‐analysis, although important limitations remain in full‐text access, quantitative chart data extraction, and expert interpretation of heterogeneity. MacAma is open‐source and available at https://github.com/YilinYuan/MacAma.

## Highlights

- We present MacAma, a large language model–driven multi‐agent framework for automated meta‐analysis.
- MacAma coordinates specialized AI agents to perform literature screening, data extraction, statistical analysis, and scientific writing.
- A dedicated Metaflow Analyzer provides automated execution and interpretation of meta‐analytic workflows.
- The framework supports transparent, reproducible, and scalable evidence synthesis through structured intermediate outputs.
- MacAma demonstrates the feasibility of AI agents acting as co‐scientists to accelerate systematic reviews and meta‐analyses.

## Graphical abstract

MacAma provides a transparent, protocol‐guided multi‐agent framework for accelerating systematic reviews and meta‐analyses. By integrating automated screening, structured extraction, quantitative synthesis, and manuscript drafting with human verification, it offers a reproducible workflow for trustworthy evidence synthesis.

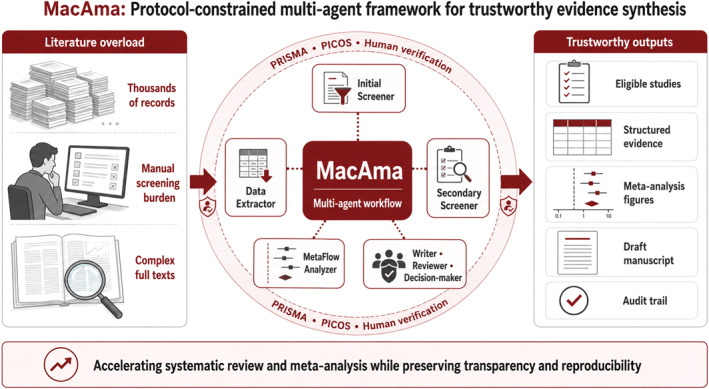

## 1 Introduction

Meta‐analysis [1] is a critical quantitative method in evidence‐based medicine, integrating data from independent studies to improve statistical power [2], resolve inconsistencies [3], and guide clinical decision‐making [3]. Synthesizing disparate findings provides a higher level of evidence than individual studies, making it indispensable for fields ranging from oncology to public health [4]. To address these systemic bottlenecks, we developed MacAma, a semi‐automated, protocol‐constrained, and human‐verifiable framework for AI‐assisted meta‐analysis. In the current case study, MacAma substantially shortened several manually intensive stages of the meta‐analysis workflow, completing the evaluated workflow within several days while preserving expert verification for high‐impact steps.

Despite its value, the traditional meta‐analysis process faces significant bottlenecks. It is inherently labor‐intensive, with the literature screening stage often identified as the most time‐consuming and bias‐prone phase [5]. Researchers must manually sift through thousands of abstracts, a process that invites subjective interpretation and fatigue‐driven errors. Furthermore, extracting granular details—such as sample sizes, experimental parameters, and outcome metrics—from vast volumes of text is inefficient and prone to omission, propagating uncertainty into the final synthesis [6].

Recent advancements have catalyzed the emergence of “AI4Research [7],” a paradigm where Large Language Models (LLMs) automate core scientific workflows. In literature retrieval, advanced artificial intelligence (AI) agents such as PaSa [8] have demonstrated the ability to traverse citation networks for deep research autonomously. However, these tools focus on relevance and broad discovery rather than the precise population, intervention, comparison, outcome, and study design ( PICOS) screening required for systematic reviews. In academic survey generation, systems such as AutoSurvey [9] and the state‐of‐the‐art SurveyForge [10] excel at generating structured, narrative‐style reviews by leveraging “scholar‐navigating intelligences.” Yet, these models remain primarily qualitative: They synthesize textual views but cannot perform quantitative data extraction and statistical synthesis, such as merging effect sizes, which are essential for meta‐analysis. In autonomous scientific discovery, frameworks such as The AI Scientist [11], and Agent Laboratory [12] have achieved end‐to‐end automation from ideation to manuscript generation. Despite their exploratory power, such fully autonomous systems may remain insufficient for high‐stakes evidence synthesis unless their outputs are constrained by explicit protocols, traceable evidence, and human verification.

A critical challenge remains: bridging the gap between automated “text generation” and rigorous “statistical evidence synthesis.” As by Lin et al. in their pivotal work “LLMs Tackle Meta‐Analysis,” [13] applying LLMs to this domain requires not just novelty but also statistical rigor and automated hypothesis verification. General‐purpose multi‐agent frameworks like AutoGen [14] or MetaGPT [15] are insufficient because scientific evidence synthesis demands alignment with methodological protocols such as Preferred Reporting Items for Systematic Reviews and Meta‐Analyses (PRISMA) [4], together with evidence‐grounded and auditable decision records. Existing “black‐box” approaches struggle to reliably parse complex multimodal data or verify extracted statistics, creating a disconnect between AI efficiency and the rigor required for high‐stakes evidence synthesis.

To bridge this gap, we propose MacAma, a semi‐automated multi‐agent framework for protocol‐constrained quantitative evidence synthesis grounded in PRISMA 2020. MacAma operationalizes PRISMA‐guided reporting requirements, PICOS‐based eligibility logic, and SYRCLE‐based risk‐of‐bias domains as concrete prompt components, decision rules, structured output fields, and audit records. In this way, each agent is not an open‐ended autonomous assistant but a specialized protocol‐executing module responsible for a defined task, such as literature screening, full‐text eligibility assessment, risk‐of‐bias evaluation, or manuscript drafting. Every AI‐generated decision is accompanied by structured rationales, predefined criteria, and traceable evidence, enabling human researchers to audit not only the final decision but also the reasoning path leading to that decision. In addition, MacAma adopts a risk‐aware human‐in‐the‐loop strategy for quantitative chart‐data extraction. Rather than pursuing full automation across all steps, MacAma delegates lower‐risk, repetitive, and protocol‐driven tasks to AI agents, while deliberately reserving high‐impact steps that directly affect effect‐size estimation and statistical conclusions for expert verification. By combining the scalability of multi‐agent systems with protocol constraints, expert‐verified data extraction, and human‐verifiable audit trails, MacAma provides a reliable pathway to accelerate evidence‐based research while preserving data fidelity and methodological traceability. Its modular design also allows potential extension to broader research communities, including medicine, psychology, and social sciences, where systematic evidence synthesis is indispensable.

The remainder of this paper is organized as follows: We first detail the methodological architecture of MacAma, explaining the five modules designed for meta‐analysis. We then present empirical results from performance benchmarks and a radiotherapy case study. Finally, we discuss implications for AI‐assisted evidence synthesis, outlining current limitations and future directions. To foster reproducibility and community collaboration, the entire MacAma framework is open‐source, with code available at https://github.com/YilinYuan/MacAma.

## 2 Methods

We developed MacAma, a semi‐automated, multi‐agent framework designed to execute high‐quality systematic reviews and meta‐analyses. Unlike generic “AI Scientist” models that aim for full autonomy, often at the cost of precision, MacAma adopts a pragmatic, human‐in‐the‐loop engineering approach. It integrates LLMs within a strict workflow to enhance efficiency while maintaining expert oversight for decisions that directly affect eligibility assessment, quantitative extraction, and statistical interpretation.

**FIGURE 1:**
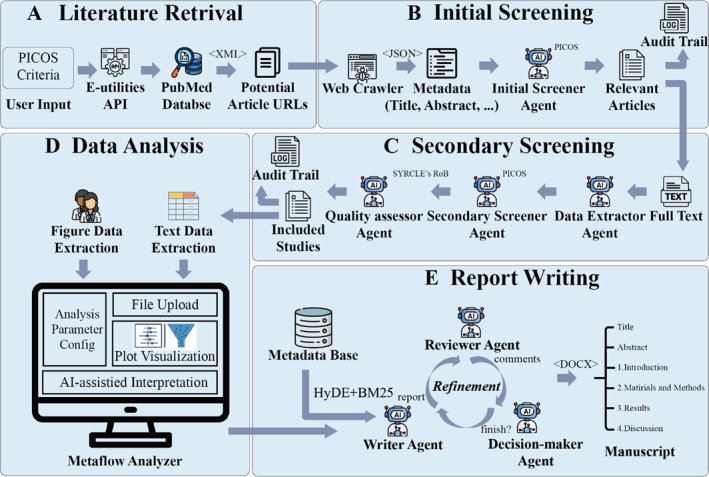
Main workflows of the MacAma. (A) Literature retrieval: Literature is retrieved from PubMed API using specified search terms based on the PICOS criteria. (B) Initial screening: an initial screening agent performs an initial screening of the literature based on titles and abstracts, which are obtained via a web crawler tool. (C) Secondary screening: A comprehensive secondary screening process evaluates full‐text articles, also acquired through the web scraper. This involves several roles: a data extractor agent, a secondary screener agent, and a quality assessor agent. (D) Data analysis: Quantitative data from the included studies were extracted using Origin and analyzed and visualized using Metaflow analyzer. This step can generate representative outputs such as forest plots, subgroup visualizations, funnel plots, pooled effect‐size estimates, heterogeneity statistics, and analysis‐ready figures, depending on the uploaded data and user‐selected parameters. (E) Report Writing: A Manuscript is generated through a collaborative multi‐agent system that includes a Writer, Reviewer, and Decision‐maker. This process utilizes the HyDE [16] and BM25 [17] algorithms, supported by RAG [18] technology.

To ensure scientific validity, the architecture of MacAma is not arbitrary; it is explicitly engineered to map the operational requirements of the PRISMA 2020 guidelines into discrete, manageable agent tasks. We decomposed the meta‐analysis pipeline into five modular workflows: literature retrieval, initial screening, secondary screening, data analysis, and report writing (Figure 1). Each module is governed by specialized agents prompted with specific protocols derived from the PRISMA 2020 guidelines. To address the “black box” issue, every decision made by an agent—whether to exclude a paper or flag a bias risk—is accompanied by a structured rationale logged in an audit trail, enabling human researchers to verify the AI's logic. Recognizing the current limitations of LLMs in handling multimodal data, particularly in extracting precise coordinates from scatterplots, we deliberately incorporated an HITL interface for data extraction, prioritizing data fidelity over full automation.

### 2.1 Protocol‐Constrained Design of MacAma

MacAma was designed as a protocol‐constrained agent framework rather than a generic multi‐agent workflow. In this design, methodological standards are not used merely as workflow references; instead, they are operationalized as explicit prompt components, decision rules, structured output fields, and audit records. PRISMA 2020 defines the overall process and reporting backbone, including literature identification, screening, eligibility assessment, synthesis, and reporting. PICOS specifies the eligibility logic used by the Initial Screener and Secondary Screener agents, including population, intervention, comparator, outcome, and study design requirements. SYRCLE's risk‐of‐bias tool provides the domain‐level assessment schema for the Quality Assessor Agent in preclinical animal studies.

Each agent is constrained to produce predefined outputs rather than free‐form judgments. For example, the Initial Screener Agent must return a document‐type judgment, semantic relevance assessment, keyword‐screening result, final inclusion/exclusion decision, and rationale. The Data Extractor Agent extracts study characteristics into predefined fields, including article information, animal model details, experimental design, detection metrics, immune‐response outcomes, and survival data. The Secondary Screener Agent then validates these structured fields against the predefined PICOS‐based inclusion and exclusion criteria. The Quality Assessor Agent produces domain‐level SYRCLE risk‐of‐bias judgments together with supporting evidence sentences and rationales. These structured outputs are retained as audit records, allowing human researchers to verify whether each automated decision follows a predefined protocol.

A representative auditable decision trail is provided in Supporting Information S1. This example links the article identifier, extracted source evidence, predefined PICOS‐based criterion, structured extraction output, agent‐generated screening decision, exclusion category, and rationale. This design allows human researchers to inspect, revise, or override agent‐generated decisions when necessary.

### 2.2 Literature Retrieval

Literature for this study was sourced primarily from the PubMed database. We automated the retrieval process using Python scripts interfacing with the National Center for Biotechnology Information (NCBI) Entrez Programming Utilities (E‐utilities) API. First, the system retrieves the PubMed IDs (PMIDs) of all potentially relevant articles. Next, it cleans the XML data returned from the E‐utilities API and extracts the corresponding PubMed URLs. Our search strategy is tailored to specific research topics, utilizing optimized keyword combinations and Boolean logic strictly in accordance with the PICOS criteria. This process efficiently generates a comprehensive list of literature URLs for subsequent screening phases.

To ensure ethical data usage, the retrieval module is strictly architected around the official E‐utilities API. The system implements rate‐limiting algorithms to comply with NCBI's server request policies (avoiding denial‐of‐service behaviors). Furthermore, for full‐text acquisition, MacAma operates within legal copyright frameworks by prioritizing open access content and strictly adhering to robots.txt protocols of publisher repositories. The system explicitly avoids unauthorized bypassing of paywalls, ensuring that data extraction respects intellectual property rights and institutional access agreements.

### 2.3 Initial Screening

The initial screening stage serves as the primary filtration gateway, designed to systematically reduce the volume of literature to a relevant and manageable subset. The process is initiated by an automated web‐crawling module that accesses each article's URL to perform programmatic data extraction. This module harvests key metadata, including the title, abstract, complete author list, publication date, document type (e.g., “Original Article”, “Review”), and keywords.

This structured metadata facilitates a two‐tier filtering process. The first tier applies a rule‐based filter to eliminate non‐original research. This step programmatically excludes document types such as reviews, meta‐analyses, commentaries, editorials and letters to the editor. In instances where the document type is ambiguous or missing, the agent analyzes the abstract's structure and linguistic cues to infer the article's nature. The second tier employs a Large Language Model (LLM) to conduct a sophisticated semantic relevance assessment, a task that goes beyond simple keyword matching. The LLM functions as a zero‐shot classifier, evaluating the title and abstract against a predefined conceptual framework. This framework is composed of essential components for the research question. By analyzing the semantic context and nuance, the model accurately identifies studies aligned with the core research objectives.

To ensure methodological transparency and support reproducible research practices, all screening decisions, along with the specific rationale and the agent responsible, are meticulously recorded in a comprehensive audit trail.

### 2.4 Secondary Screening

Articles that successfully pass the initial screening are subjected to a rigorous full‐text appraisal during the secondary screening stage. This phase ensures that the remaining studies not only are relevant but also meet the stringent methodological criteria required for data synthesis and meta‐analysis.

The workflow commences with the full‐text acquisition from the previously identified URLs. A specialized Data Extractor Agent performs targeted content parsing, focusing specifically on the “Methods” and “Results” sections of each paper. This agent is engineered to identify and extract granular details, including specific animal model parameters (strain, age, and sex), experimental designs (e.g., control and treatment arms and sample sizes), intervention specifics (e.g., irradiation dosage and fractionation schedule), and a predefined list of immunological indicators and outcome measures. Following extraction, a Secondary Screener Agent validates this structured data against a predefined inclusion and exclusion protocol. This validation is a critical quality control step, confirming, for example, that the study is an in vivo experiment, that it includes a valid comparator or control arm, and that it reports quantitative data on the effects of x‐ray irradiation on relevant immune indicators.

Any study failing to meet one or more of these criteria is excluded. The specific reason for exclusion is automatically documented, which is essential for generating PRISMA‐compliant flow diagrams. This meticulous two‐agent process guarantees that only high‐quality, methodologically sound studies proceed to the final stages [19].

### 2.5 Data Extraction and Analysis

MacAma incorporates a risk‐aware human‐in‐the‐loop workflow for extracting quantitative data [20] from scientific figures. In meta‐analysis, numerical values extracted from plots, including group means, error bars, sample sizes, and survival‐related coordinates, directly determine effect‐size estimation and downstream statistical conclusions. We therefore deliberately reserve this high‐impact step for domain researchers using professional graphing software such as Origin, while delegating lower‐risk, repetitive, and protocol‐driven tasks, including literature screening, structured text extraction [21], and preliminary bias assessment, to AI agents. This design prioritizes data fidelity and statistical validity over indiscriminate full automation. Using Origin, domain researchers extracted X and Y coordinate values, associated error bar values, and in‐figure sample size information. These extracted values were manually checked before analysis and then uploaded to Metaflow Analyzer for statistical synthesis and visualization.

All verified data are subsequently uploaded to Metaflow Analyzer, an interactive web‐based platform built on Streamlit. This application is designed to democratize advanced statistical analysis and features a modular architecture:

Data Upload and Integration: Users can upload standardized files (such as Excel or CSV) through an intuitive interface. The platform provides an intuitive user interface for uploading standardized data files (e.g., Excel or CSV), which are parsed and integrated into a unified analytical dataset [22].

Parameter Configuration: Users are afforded complete control over the analysis, with options to select the type of effect size (e.g., Hedges' g, Odds Ratio), the statistical model (Fixed‐Effect or Random‐Effects), and the tests for statistical heterogeneity.

Backend Analytical Engine: A robust backend, leveraging powerful Python libraries, automatically executes all statistical computations upon parameter selection.

Dynamic Visualization: The platform dynamically generates a suite of analysis‐ready visualizations, including forest plots to display individual and pooled effect sizes, and funnel plots to assess potential publication bias.

AI‐Assisted Interpretation: To bridge the gap between quantitative output and qualitative understanding, the web provides AI‐generated narrative summaries of the results. It synthesizes key statistical outputs (e.g., pooled effect size, 95% confidence intervals, *p*‐value, and the I^2^ statistic for heterogeneity) [23] into explanatory text, helping users to interpret the analytical findings rapidly.

This platform effectively synergizes interactive user control with a powerful and automated analytical engine, streamlining the entire meta‐analysis process.

**FIGURE 2:**
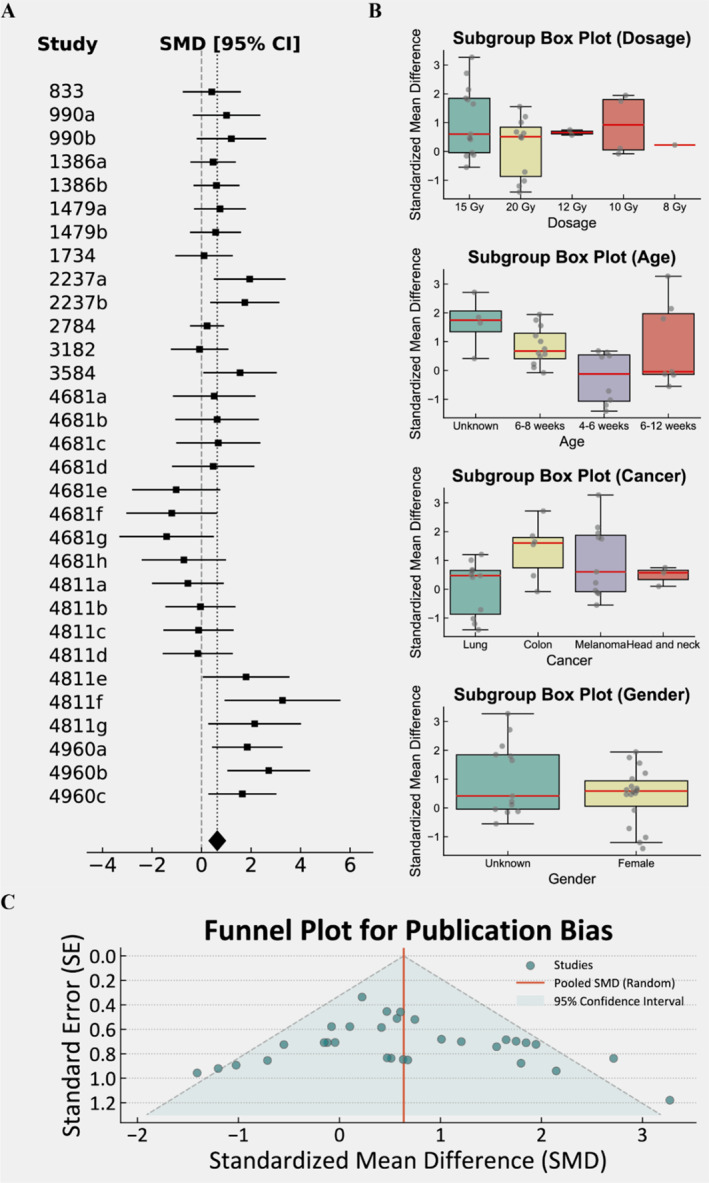
Meta‐analysis using Metaflow Analyzer. (A) Forest plot of SMD and 95% CI. (B) Subgroup box plots. (C) Funnel plot for bias.

In the current case study, the Data Analysis step corresponds to the analyses and visualizations shown in Figure 2. After quantitative values were organized into standardized input files, Metaflow Analyzer calculated study‐level standardized mean differences (SMDs), pooled estimates, 95% confidence intervals, and heterogeneity statistics. It also generated representative visual outputs, including forest plots, subgroup visualizations, and funnel plots. Figure 2 presents representative outputs from this Data Analysis step rather than an exhaustive list of all possible outputs. Depending on the uploaded data and user‐selected parameters, this step can additionally export study‐level effect‐size tables, pooled SMD summaries, heterogeneity statistics, subgroup summaries, and analysis‐ready figures. Funnel plots were used for visual assessment of potential reporting bias, whereas heterogeneity and subgroup findings were interpreted with expert caution.

### 2.6 Report Writing

The final stage of MacAma generates a complete scientific manuscript through a structured multi‐agent workflow. In contrast to conventional one‐shot generation, which can introduce hallucinations and disrupt logical continuity, MacAma implements a collaborative multi‐agent refinement loop (Figure 1) that mirrors the iterative drafting and review practices of a research team. This module consists of three specialized agents: the Writer, Reviewer, and Decision‐Maker—operating within a predefined algorithmic pipeline.

Prior to drafting, the system establishes a grounded knowledge base to minimize unsupported inferences. To narrow the semantic gap between a concise user‐provided topic and domain‐specific scientific language, we apply the hypothetical document embeddings (HyDE) [16] approach. The model first produces a hypothetical abstract that enhances the query with relevant terminology. This enriched query is then used to search the local knowledge base, indexed during the screening phase, using the Best Matching (BM25) [17] algorithm. In the current case study, the top 20 relevant citations were retrieved and supplied to the Writer Agent at each drafting stage. This value was fixed across drafting stages to ensure a consistent evidence base during manuscript generation.

The manuscript generation process follows an iterative self‐correction cycle: in the first stage of the refinement cycle, the Writer Agent synthesizes the research topic, the structured outputs from the Analysis module, and the retrieved citations to generate an initial manuscript draft that tightly adheres to the available evidence. This draft is then passed to the Reviewer Agent, who conducts a critical evaluation focusing on logical consistency, accuracy of interpretation, citation correctness, and alignment with academic writing standards. The reviewer provides structured feedback that highlights specific issues, such as discrepancies between the narrative and the underlying data. Finally, the Decision‐Maker Agent assesses the reviewer's comments and issues a binary judgment—NEEDS_REVISION or LOOKS_GOOD. Drafts deemed to require further refinement are returned to the Writer Agent for targeted revisions, whereas those assessed as satisfactory progress to the next stage of the workflow.

A maximum of three refinement rounds is permitted to prevent indefinite cycling. This iterative review loop generates evidence‐grounded and logically coherent manuscript drafts, thereby reducing the amount of initial manual drafting and revision typically required. However, the generated manuscript draft is not treated as publication‐ready. Human researchers remain responsible for verifying extracted evidence, checking statistical interpretation, revising scientific arguments, correcting factual errors, and approving the final manuscript.

### 2.7 Benchmark Construction and Case‐Study Reproducibility

For the full‐text secondary‐screening benchmark, 599 articles were selected from the 749 obtainable full‐text articles because they contained complete full‐text content and complete human annotation required for evaluation. The selection was therefore based on data completeness rather than model performance. None of the 599 benchmark articles was used for prompt development or refinement; prompt development was conducted using a separate pilot set excluded from the final benchmark evaluation.

Two human annotators independently screened the benchmark records according to the predefined PICOS‐based eligibility criteria and were blinded to model‐generated labels during annotation. Disagreements were resolved through discussion, and unresolved cases were adjudicated by a senior reviewer. The resulting consensus labels were used as the gold‐standard reference for evaluating the screening agents.

The objective of the radiotherapy case study was to evaluate preclinical evidence on how x‐ray radiotherapy modulates anti‐tumor immune responses in animal tumor models. The standardized inquiry was formulated as follows: in vivo mouse tumor models, compared with non‐irradiated controls, how does x‐ray irradiation affect tumor‐related immune outcomes, including immune‐cell infiltration, immune‐checkpoint expression, and cytokine expression? The corresponding PICOS‐based criteria, exact Boolean search strategy, structured input fields, screening records, analysis files, and figure generation data are provided in the case study folder of the GitHub repository, where they are legally shared.

### 2.8 Use of AI Tools and Human Accountability

MacAma and related AI tools were used to support literature screening, structured extraction, preliminary risk‐of‐bias assessment, statistical workflow organization, and manuscript drafting. These tools were not treated as authors because they cannot take responsibility for the integrity, interpretation, or accountability of the work. Human researchers retained responsibility for study design, eligibility criteria, data extraction decisions, statistical interpretation, manuscript revision, and final approval. The role of AI tools, including tool names, versions when available, workflow functions, and the extent of human verification, should be transparently disclosed in the Methods, Acknowledgments, or a dedicated AI‐use statement.

### 2.9 Overview

Our work ensures efficient task scheduling and data flow, offering greater flexibility compared to traditional tools. The detailed prompts used to guide the AI agents for initial screening, secondary screening, data extraction, and risk of bias assessment are provided in the (Supporting Information S1: Figure S1–S6).

## 3 Results

MacAma repositions part of the researcher's role from manual execution of repetitive screening and extraction tasks toward supervision, verification, and interpretation within a protocol‐constrained workflow. This paradigm shift allows experts to focus their efforts on higher‐level analysis, interpretation of synthesized data, and handling the nuanced edge cases identified by the AI.

To validate MacAma's accuracy, we benchmarked its performance against human consensus reference labels generated by two independent human annotators. Furthermore, we conducted comprehensive ablation studies to isolate the contribution of our structured prompt engineering design from the inherent capabilities of the underlying LLM.

### 3.1 Benchmark Evaluation

To evaluate the screening performance of MacAma at different stages, we constructed separate benchmark datasets for the initial title/abstract screening task and the full‐text secondary screening task.

For the initial screening benchmark, a manually labeled set of 354 records was used to evaluate title/abstract‐level screening performance. These records were labeled according to the predefined eligibility criteria and were used to compare MacAma with baseline models and ablation settings. The benchmark records used for this evaluation were not used for prompt development or refinement.

For the full‐text secondary screening benchmark, 749 full‐text articles were obtainable from the 1298 records retained after initial screening. Among these, 599 articles with complete full‐text records and complete human reference labels were included in the benchmark evaluation. The selection of the 599 benchmark articles was therefore based on the availability of complete full‐text content and complete human annotation, rather than model performance.

None of the 599 benchmark articles was used for prompt development or refinement. Prompt development was conducted using a separate pilot set that was excluded from the final benchmark evaluation.

Two human annotators independently screened the benchmark records according to the predefined PICOS‐based eligibility criteria. The annotators were not exposed to the model‐generated labels during annotation. Disagreements were resolved through discussion, and when consensus could not be reached, a senior reviewer made the final decision. The consensus labels were used as the gold‐standard reference for evaluating the screening agents.

**FIGURE 3:**
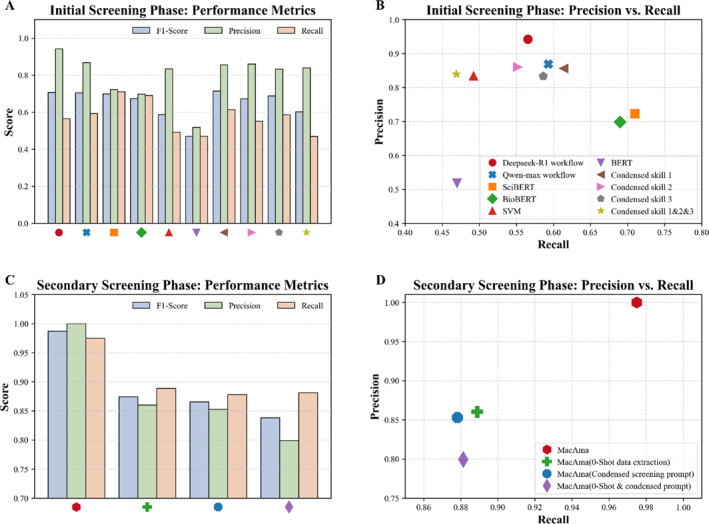
Comparative analysis of screening performance. (A) Performance metrics (F1‐Score, Precision, Recall) for the Initial Screening Phase across different models and ablation settings. (B) Precision versus Recall landscape for initial screening. The DeepSeek‐R1 workflow achieves high precision with competitive recall. (C) Performance metrics for the Secondary Screening Phase, highlighting the impact of prompt engineering and data extraction strategies. (D) Precision versus Recall for secondary screening, where the full MacAma implementation achieves near‐perfect classification (top‐right corner).

Initial Abstract Screening: In the crucial initial screening phase, which involved 354 abstracts, the workflow powered by the DeepSeek‐R1 model [24] demonstrated superior performance, distinguishing itself as the most efficient tool for researcher‐supervised screening (Figure 3A). As shown in Table 1 and Figure 3B, the DeepSeek‐R1 Workflow achieved a leading Precision of 94.25%, outperforming both the robust Qwen‐max [25] baseline (86.87%) and traditional domain‐specific encoders like SciBERT (72.24%) and BioBERT (69.80%). The full workflow achieved the highest precision and lowest NNR among the compared settings, whereas a condensed variant achieved a slightly higher F1 by increasing recall at the cost of precision. Most notably, our framework attained an exceptional Number Needed to Read (NNR) of 1.0610. This NNR value—approaching the theoretical ideal of 1.0—indicates that for every document the system flagged as relevant, nearly every single one was indeed a valid hit. In contrast, traditional methods like BERT required reading nearly twice as many documents (NNR 1.93) to find a relevant one. This high‐precision characteristic validates MacAma's ability to significantly reduce the cognitive load on researchers, supporting high‐precision prioritization while requiring human oversight or sensitivity checks to avoid missing eligible studies.

**TABLE 1:** Performance comparison of different methods during the screening task.

Method	F1‐score	Precision	Recall	NNR
DeepSeek‐R1 workflow	0.7069	94.25%	56.55%	1.0610
Qwen‐max workflow	0.7049	86.87%	59.31%	1.6862
SciBERT	0.6988	72.24%	71.00%	1.4080
BioBERT	0.6742	69.80%	69.00%	1.4549
SVM	0.5875	83.36%	49.22%	1.2550
BERT	0.4705	51.82%	47.00%	1.9298
DeepSeek‐R1 Workflow (condensed skill 1)	0.7149	85.58%	61.38%	1.1685
DeepSeek‐R1 Workflow (condensed skill 2)	0.6723	86.02%	55.17%	1.1626
DeepSeek‐R1 Workflow (condensed skill 3)	0.6883	83.33%	58.62%	1.2000
DeepSeek‐R1 Workflow (condensed skill 1& skill 2 & skill 3)	0.6018	83.95%	46.90%	1.1912

*Note:* This table presents F1‐Score, Precision, Recall, (NNR), and ablation studies of the DeepSeek‐R1 Workflow with condensed skills.

Full‐Text Secondary Screening: In the full‐text screening stage, we deployed Qwen‐Max as the Data Extractor Agent and DeepSeek‐R1 as the Secondary Screener Agent. MacAma's performance at this stage was powerful. When validated against 599 “gold‐standard” full‐text articles, it achieved an F1‐score of 0.9873 for inclusion and exclusion decisions (Figure 3C). As shown in Table 2, this performance was supported by a flawless 100% precision rate, indicating that every article selected for inclusion was correct—eliminating all false positives from the constructed 599‐article benchmark. Combined with a recall of 97.49%, MacAma achieved both high accuracy and comprehensive coverage, occupying the optimal position in the precision‐recall landscape (Figure 3D).

**TABLE 2:** Comparative ablation results for the full‐text secondary screening module.

Method	Accuracy (%)	Precision (%)	Recall (%)	F1‐score	Sample size
MacAma	98.83	100	97.49	0.9873	599
MacAma (0‐shot data extraction)	88.48	86.02	88.89	0.8743	599
MacAma (condensed screening prompt)	87.65	85.3	87.82	0.8655	599
MacAma (0‐shot data extraction & condensed screening prompt)	85.64	79.93	88.14	0.8383	599

To assess the effectiveness of our prompt design, we conducted ablation experiments across both stages of the screening process. Specifically, we evaluated the contribution of the three reasoning components defined in the “Literature Screening” system prompt (Supporting Information S1: Figure S1): (1) Document Type Check, (2) Semantic Reasoning Analysis, and (3) Keyword Screening. Performance of the full MacAma protocol was compared with simplified variants in which these steps were removed or merged (Figure 3A). The un‐ablated, multi‐step prompt consistently achieved higher precision, whereas omitting the semantic reasoning or keyword verification components resulted in a substantial decline in screening accuracy (Figure 3B). These findings indicate that hierarchical task decomposition is critical for reliably excluding irrelevant literature.

We further examined the prompts used by the Data Extractor and Secondary Screener agents. A structured extraction prompt was evaluated against a zero‐shot approach without schema constraints. The zero‐shot method frequently failed to capture detailed study attributes—for instance, specific irradiation fractionation—whereas the structured prompt maintained high data fidelity (Figure 3C). We also tested a simplified version of the inclusion/exclusion logic prompt. The complete prompt, which explicitly enforces logical validation against PICOS criteria, demonstrated superior accuracy relative to the condensed form (Figure 3D). Collectively, these ablation experiments demonstrate that MacAma's performance gains stem not only from the capabilities of the underlying model but also critically from the carefully engineered, step‐by‐step reasoning framework embedded within its agent architecture.

### 3.2 Case‐Study Workflow Performance

To illustrate MacAma's operational mechanics and demonstrate its real‐world efficacy, a case study was executed focusing on the interplay between radiotherapy and anti‐tumor immunity. MacAma was configured with a search window from January 1, 2010 to February 8, 2025. A complex Boolean search string was designed to capture preclinical studies involving tumors, x‐ray therapy, and associated immune responses in animal models. Our automated script programmatically queried the PubMed API, retrieving an initial corpus of 5598 articles. This corpus then served as the input for the multi‐agent screening and analysis pipeline.

The standardized inquiry for this case study was: in vivo mouse tumor models, compared with non‐irradiated controls, how does x‐ray irradiation affect tumor‐related immune outcomes, including immune‐cell infiltration, immune‐checkpoint expression, and cytokine expression? The corresponding PICOS‐based eligibility criteria, Boolean search strategy, structured input fields, screening records, analysis files, and figure generation data are provided in the case study repository, where they are legally shareable.

#### 3.2.1 Literature Retrieval and Screening

**FIGURE 4:**
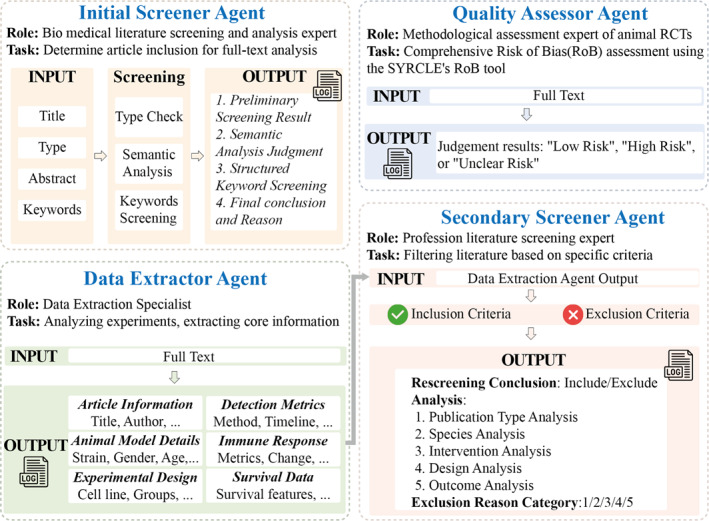
Settings of screening agents. Initial screener classifies via abstract/metadata; Data extractor parses full text; Secondary screener validates; Quality assessor uses SYRCLE's RoB.

The process begins with the Literature Retrieval module, which parses the initial XML‐formatted list of PubMed IDs, retrieves the corresponding publication URLs, and prepares the articles for screening. As depicted in Figure 4, MacAma is founded on a sequence of specialized intelligent agents that collaborate to refine the initial literature corpus. The first layer of analysis is managed by the Initial Screener Agent (Supporting Information S1: Figure S1), which functions as an expert in biomedical literature classification. Tasked with determining if a document warrants full‐text review, this agent processes the abstract, document type, title, and keywords through a structured “Literature Screening” skill. Articles passing this initial validation proceed to a more rigorous evaluation by a duo of agents. The Data Extractor Agent, acting as a scientific analysis expert, meticulously parses the full text to extract and structure key information into predefined categories: Article Information, Animal model details, Experimental design, Detection metrics, Immune response, and Survival data (Supporting Information S1: Figure S3). This structured output is then passed to the Secondary Screener Agent (Supporting Information S1: Figure S4). This agent functions as a protocol enforcement expert, cross‐validating the extracted details against the specific inclusion and exclusion criteria of the study protocol, ensuring that every article proceeding to analysis is fully compliant. Included articles will also undergo bias risk analysis by a quality assessor agent (Supporting Information S1: Figure S6).

To illustrate the auditability of this screening process, we provide a representative full‐text secondary screening record in Supporting Information S1. This record contains the complete structured output generated by the Data Extractor Agent and the subsequent conclusion, inclusion reason, and reasoning process generated by the Secondary Screener Agent. It shows how a full‐text screening decision can be traced from extracted study information to predefined PICOS‐based eligibility criteria and then to the final inclusion or exclusion judgment. Such decision trails allow human researchers to inspect the extracted evidence and reasoning chain, thereby supporting verification, revision, or override of agent‐generated decisions when necessary.

**FIGURE 5:**
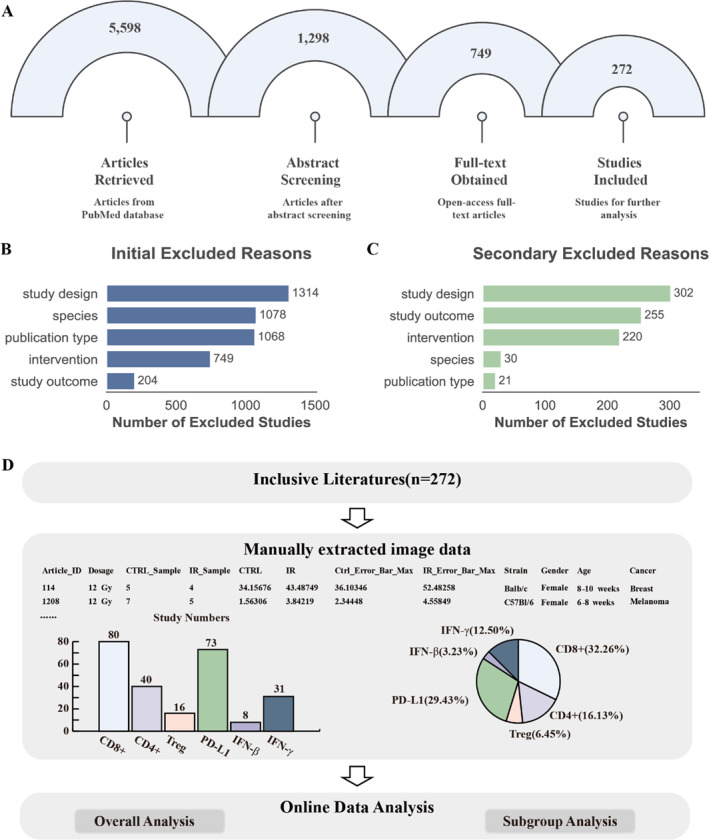
Literature screening and data synthesis. (A) Flowchart showing progression from 5598 retrieved articles to 272 included studies. (B) Initial exclusion reasons. (C) Secondary exclusion reasons. (D) Synthesized data distribution from included studies.

Following the retrieval, the 5598 articles sourced from the PubMed database underwent a rigorous, multi‐stage filtering process, as illustrated in Figure 5A. The initial phase involved an AI‐driven abstract screening. This step significantly narrowed the field by eliminating 4300 irrelevant papers, after which 1298 articles were retained for further consideration.

The subsequent stage involved acquiring the full text of the remaining articles. Open‐access full texts were successfully obtained for 749 of these articles. These then proceeded to a detailed full‐text screening to verify that they met all inclusion criteria. This intensive final review resulted in the exclusion of 477 more articles, yielding a final cohort of 272 studies deemed suitable for further analysis. The reasons for excluding articles were documented and categorized into five groups, which are shown in Figure 5B,C.

#### 3.2.2 Data Extraction and Analysis

For the final 272 included articles, MacAma facilitated an in‐depth data extraction process to capture essential experimental details (Figure 5D). This yielded a rich, structured dataset including specific irradiation dosages, sample sizes for both control and intervention groups, and granular animal model information such as strain (e.g., Balb/c, C57BL/6), gender, and age. The synthesis of the data revealed a broad research landscape, quantifying the number of studies that reported on key immune markers, including CD8+ T cells (80 studies), CD4+ T cells (40 studies), Treg cells (16 studies), and the immune checkpoint molecule PD‐L1 (73 studies), IFN‐γ (31 studies), IFN‐β (8 studies).

This compiled dataset enabled a comprehensive meta‐analysis, supported by MacAma's interactive “zero‐code” analysis platform. This platform empowers researchers without expertise in statistical programming languages (like R or Stata) to conduct sophisticated analyses. Through an intuitive graphical interface, users can compute pooled effect sizes, perform subgroup analyses, and assess publication bias using dynamically generated visualizations such as forest plots and funnel plots. This democratization of advanced statistical methods significantly lowers technical barriers and allows researchers to focus on the scientific interpretation of findings.

Representative outputs of the Data Analysis step are presented in Figure 2. Figure 2A shows the forest plot generated by Metaflow Analyzer, including study‐level and pooled SMD estimates with 95% confidence intervals. Figure 2B presents subgroup visualizations used to explore potential sources of heterogeneity across predefined study‐level variables. Figure 2C shows the funnel plot generated for visual assessment of potential reporting bias.

In summary, these findings not only address key scientific questions but also validate MacAma as an efficient and reliable tool for rigorous, evidence‐based research [26]. See Supporting Information S1: Figure S7–S11 for comprehensive data analysis [27].

#### 3.2.3 Quality Assessment

A novel feature of MacAma is the automation of the SYRCLE risk of bias assessment for preclinical studies [28]. For each of the 272 included articles, MacAma programmatically scanned the text for keywords related to established bias domains (e.g., “random”, “blinded”, “allocation”). It then extracted the relevant sentences as evidence and proposed a preliminary judgment (“Low Risk”, “High Risk”, or “Unclear Risk”) with a supporting rationale. For example, if an article failed to describe its randomization method, the agent would flag “Sequence Generation” as an “Unclear Risk” and generate the rationale: “The method for generating the random sequence was not clearly described in the article.” This feature empowers the researcher to make a swift, evidence‐based final decision, drastically accelerating the quality assessment process [29].

**FIGURE 6:**
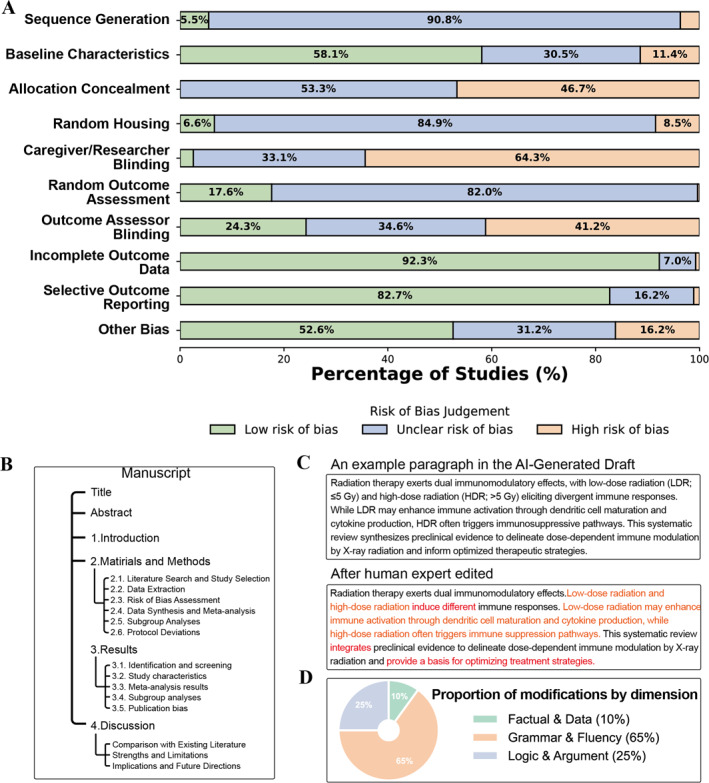
Bias assessment and manuscript evaluation. (A) Bias summary. (B) AI manuscript structure. (C) Editing example. (D) Revision proportions.

The aggregated results of this automated assessment across all included studies are summarized in Figure 6A. This meta‐level overview revealed common reporting deficiencies within the field. While a high percentage of studies demonstrated a low risk of bias for reporting complete outcome data (92.3%) and for avoiding selective outcome reporting (82.7%), significant gaps were identified elsewhere. A vast majority of studies presented an “Unclear Risk” of bias regarding the blinding of investigators (84.9%) and outcome assessors (64.3%), highlighting a critical area for improvement in the methodological reporting of preclinical radiotherapy research.

#### 3.2.4 Automated Manuscript Generation

After the analysis is complete, MacAma's writing workflow generates a complete first draft of the manuscript. The AI composes a well‐structured document that includes all standard sections, such as Introduction, Methods, Results and Discussion, as outlined in Figure 6B. This output should be interpreted as a draft for expert revision rather than a publication‐ready manuscript.

To provide a preliminary description of the revision burden associated with MacAma‐generated drafting, we analyzed the manual edits made to the AI‐generated manuscript draft in the current radiotherapy case study. This analysis was based on a single manuscript draft and categorized edits at the sentence or local paragraph level according to their primary purpose: grammar/prose refinement, logic/argumentation improvement, or factual/data correction. As an illustrative example, Figure 6C shows one sentence‐level before‐and‐after revision. As shown in Figure 6D, most recorded edits were related to grammar and prose refinement (65.0%), followed by logic and argumentation improvement (25.0%) and factual or data correction (10.0%). These results should be interpreted as a preliminary single‐case estimate of the revision burden rather than as a systematic evidence of manuscript‐level quality across studies.

### 3.3 Overall Efficiency Gains

The cumulative impact of MacAma from screening and extraction to analysis and writing *suggests substantial workflow‐level efficiency gains in the current radiotherapy case study*. The most transformative results are seen in the compression of the research timeline. As summarized in Table 3, a systematic review is completed substantially faster than the estimated traditional manual workflow in this case study.

**TABLE 3:** Time comparison between MacAma and the traditional process.

Stage	Traditional manual	MacAma	Efficiency gain
Initial screen (5,598)	9–12 months	∼56 h	∼130x speedup
Secondary screen (749)	6–8 months	∼24 h	∼200x speedup
Analysis & plotting	1–3 h (incl. code)	∼10 min	∼10x speedup
Total (excl. writing)	> 1.5 years	< 4 days	> 150x speedup

Initial Screening: What typically takes 9–12 months of dedicated human effort to screen 5598 articles was completed by MacAma in approximately 56 h.

Secondary Screening: The subsequent full‐text review of 749 articles, a task that consumes another 6–8 months, was finished in roughly 24 h.

Analysis and Plotting: The final stage of data analysis, which can take hours or even days of coding and computation, was accomplished in less than 10 min.

In total, the key process for this systematic review, a task that traditionally took over 1.5 years, was completed in less than 4 days with MacAma. These estimates should be interpreted as approximate workflow‐level comparisons for the current case study, rather than generalizable time‐saving guarantees across all meta‐analyses. The results support MacAma as a promising protocol‐constrained framework for reducing repetitive workload while preserving human oversight for eligibility decisions, quantitative data extraction, statistical interpretation, and manuscript revision.

## 4 Discussion

### 4.1 Key Findings and Contributions

This study presents and evaluates MacAma, a semi‐automated meta‐analysis framework that integrates LLMs and multi‐agent systems. By operationalizing selected PRISMA 2020 reporting items, PICOS‐based eligibility logic, and SYRCLE‐based risk‐of‐bias domains as structured prompts, decision rules, output fields, and audit records, MacAma showed substantial workflow‐level efficiency gains in the current radiotherapy case study, while maintaining a protocol‐constrained and human‐verifiable workflow.

The distinction between MacAma and existing AI4Research systems is primarily task‐specific rather than purely performance‐based. The AI Scientist and Agent Laboratory are designed for open‐ended scientific discovery, AutoSurvey and SurveyForge for narrative survey generation, and PaSa [8] for relevance‐driven academic search. By contrast, MacAma is designed for quantitative evidence synthesis under explicit evidence‐synthesis constraints. Its workflow embeds PICOS‐based eligibility logic, SYRCLE‐based risk‐of‐bias assessment, structured data extraction, statistical synthesis, and auditable decision records into a meta‐analysis pipeline. Thus, MacAma should be viewed as a protocol‐constrained framework for semi‐automated meta‐analysis rather than a general‐purpose autonomous research agent.

The core contribution of MacAma lies in harnessing advanced AI technologies to surmount key efficiency bottlenecks in traditional meta‐analysis, thereby proposing a novel paradigm for future research automation. Rather than claiming full automation of the systematic review process, MacAma follows a risk‐aware automation strategy: lower‐risk, repetitive, and protocol‐driven tasks are delegated to AI agents, whereas steps that directly influence effect‐size estimation, statistical conclusions, and final scientific interpretation remain subject to human verification. This approach not only streamlines processes within AI and meta‐analysis domains but also extends its generality and scalability to broader research communities, including medicine, psychology, and social sciences, where systematic literature reviews are essential for evidence‐based inquiry.

First, this study improves the efficiency and consistency of literature screening in the evaluated workflow, traditionally the most labor‐intensive and subjectivity‐prone phase of meta‐analysis. Conventional methods often require researchers to manually sift through thousands of articles over months or even years, introducing substantial biases and delays [30]. MacAma's LLM‐based intelligent screening agent accelerates this process dramatically—as demonstrated in the case study, compressing the entire cycle from years to mere days. Experimental validation further corroborates its efficacy, with a precision of 94.25% in the initial screening phase and 100% in the secondary phase, underscoring MacAma's ability to uphold high scientific standards while boosting productivity. This human‐AI collaborative model liberates researchers from rote tasks, enabling them to concentrate on strategic decision‐making and oversight, thereby delivering tangible practical value—particularly for high‐volume literature reviews in fields like medicine and social sciences [31]. These findings should be interpreted as evidence of improved efficiency within the evaluated case‐study workflow, rather than as a universal guarantee of time savings across all meta‐analyses.

Second, at the architectural level, MacAma validates a scalable “collaborative intelligence” paradigm. Consistent with recent trends in Agent Laboratory and multi‐agent collaboration, we move away from monolithic LLM calls toward a modular, role‐based system. By encapsulating tasks—screening, extraction, analysis—into independent units, MacAma minimizes the “error propagation” often seen in single‐agent workflows. This “soft‐coded” design allows for the seamless integration of future, more capable models without architectural overhaul, ensuring the framework's longevity.

In this case study, MacAma provides semi‐automated support across multiple stages, while preserving human verification for high‐impact steps. This lays a robust foundation for the next generation of “AI Science Assistants” [32]. Overall, these contributions alleviate persistent challenges in automated scientific discovery, paving the way for future AI integrations that emphasize standardization, multimodal proficiency, and cross‐disciplinary utility, ultimately empowering diverse scientific communities to advance evidence‐based research with greater efficiency and reproducibility.

### 4.2 Limitations and Challenges

Despite these advancements, MacAma faces constraints that reflect the current boundaries of AI4Research: Dependency on HITL for Multimodal Extraction: As noted in benchmarks like ChartQA [33] and ChartX [34], current multimodal LLMs still struggle with the precise extraction of numerical data from complex scientific figures. Consequently, MacAma deliberately retains an HITL interface for chart data. While the AI Scientist attempts full automation, we posit that for evidence‐based medicine, retaining human verification of visual data is currently a necessary safeguard for validity [35]. This design should not be viewed merely as a workaround for model limitations; rather, it reflects a risk‐aware automation strategy in which high‐impact steps that directly affect effect‐size estimation and statistical conclusions remain subject to expert verification.

Sensitivity to Retrieval Quality: The system's output quality is bound by its input. While MacAma optimizes screening, it relies on external databases. Future iterations could benefit from integrating Deep Research strategies (similar to PaSa [8]) to support broader and a more adaptive search‐query expansion beyond user‐provided keywords. However, such expansion should remain transparent, versioned, and auditable, because changes in the search strategy can affect study selection and reproducibility.

Heterogeneity interpretation: Although MacAma can automatically calculate heterogeneity indicators (like I^2^) and perform predefined subgroup analyses, it cannot yet deeply understand and interpret potential sources of heterogeneity like a human expert [36] or propose new subgroup or meta‐regression hypotheses based on data characteristics. This limits its intelligence in exploratory analysis scenarios. As suggested by Lin et al. [13], automating the explanation of statistical variance remains a frontier challenge that requires integrating causal reasoning models. Therefore, heterogeneity statistics and subgroup results generated by MacAma should be treated as decision‐support outputs rather than autonomous scientific conclusions. Expert interpretation remains essential, especially when subgroup analyses are exploratory, underpowered, or affected by differences in tumor model, irradiation dose, measurement method, or sampling time point.

Limited full‐text access and potential sample bias: In the case study, due to paywall restrictions, the actual number of obtainable full‐text articles was only about 58% (749/1298) of the literature that passed the initial screening [37]. This access limitation may introduce selection bias, thereby affecting the representativeness and robustness of the final meta‐analysis results. Therefore, how to integrate with institutional knowledge bases to break down access barriers is a major challenge for improving usability and analytical accuracy [38]. MacAma does not bypass paywalls, publisher access controls, robots.txt restrictions, or institutional license limitations; future integration with institutional library systems should comply with publisher licenses, institutional policies, copyright regulations, and applicable regional legal frameworks.

Benchmark and drafting generalizability: The current evaluation is based on one preclinical radiotherapy case study and task‐specific benchmark datasets. Although the results support the feasibility of MacAma in this setting, its performance across other biomedical topics, clinical systematic reviews, non‐biomedical disciplines, and different evidence structures remains to be established. In addition, the manuscript generation analysis was based on a single case study draft and should be interpreted as a preliminary description of the revision burden rather than as a systematic evidence of publication‐level writing quality across topics.

Human annotation reliability: The screening benchmarks relied on human consensus labels. If formal inter‐annotator agreement was not calculated, this should be explicitly acknowledged as a limitation; if calculated, the corresponding Cohen's *κ* or percent agreement should be reported. This information is important for interpreting model performance relative to human agreement and for assessing the reliability of the reference labels.

### 4.3 Future Research Directions

To advance the field of automated evidence synthesis, future work [39] should focus on the following:

Continuous breakthroughs in multimodal parsing capabilities: Integrating specialized vision‐language models fine‐tuned on scientific figures to automate the “last mile” of data extraction, reducing reliance on manual HITL steps.

Deep integration with institutional knowledge bases: Develop legal API interfaces with university and research institution library subscription systems, enabling MacAma to access more paywalled full‐text resources through institutional permissions, thereby significantly improving data completeness and the credibility of analysis results. Such integration should be implemented only through approved APIs or authorized library services and should not be framed as bypassing access barriers.

Enhancement of interactive guidance and explainability [40]: Further optimize the human‐computer interaction process, explore the functionality for MacAma to automatically diagnose and provide intelligent suggestions on user input, and provide more transparent and traceable decision‐making evidence at key stages like screening and exclusion, to enhance user trust and control over output. Future versions could also provide uncertainty indicators, side‐by‐side displays of source evidence and agent decisions, and clearer alerts when PICOS criteria or extracted fields are incomplete or ambiguous.

Self‐Correction Mechanisms: Implementing iterative “self‐reflection” loops similar to SurveyForge's [10] refinement process allows agents to cross‐check extracted data against the original text to further suppress hallucinations [41]. These mechanisms should be designed to strengthen auditability and consistency rather than to remove human oversight.

From Meta‐Analysis to “Living Reviews”: Evolving MacAma into a dynamic system that continuously monitors new literature and automatically updates meta‐analyses [42], with versioned search strategies, transparent update logs, and reproducible records of how newly included studies change pooled estimates or conclusions.

Evolution toward a broader “AI Science Assistant”: Practically, future work can adopt a standardized, model‐agnostic tool‐interface protocol (e.g., the Model Context Protocol, MCP) as the common tool layer for AI scientists, enabling extensions to higher‐level tasks such as automated experimental design, complex data analysis, and hypothesis generation, and gradually building an “AI Scientist” with partial independent research capabilities [43] to scale and accelerate research. Nevertheless, for high‐stakes scientific claims, MacAma and related systems should function as auditable assistants rather than autonomous replacements for human researchers.

## 5 Conclusion

We introduce MacAma, a semi‐automated framework that synergizes large language models and multi‐agent systems to address the labor intensity and bias of traditional meta‐analysis. By embedding protocol constraints, structured outputs, human‐verifiable audit records, and risk‐aware human‐in‐the‐loop verification into the workflow, MacAma supports AI‐assisted quantitative evidence synthesis while preserving expert oversight for high‐impact steps. In the current case study, MacAma substantially shortened several manually intensive workflow stages from estimated manual timescales of months to automated processing times of days, while maintaining a protocol‐constrained and human‐verifiable workflow.

The core contribution of MacAma is not merely the use of multiple AI agents, but the construction of a protocol‐constrained, auditable, and modular workflow for quantitative evidence synthesis within the AI4Research landscape. Empirical validation showed competitive screening performance in the evaluated benchmark and substantial workflow‐level efficiency gains in the current radiotherapy case study. On a theoretical level, it supports the feasibility of a “soft‐coded” automation paradigm based on MAS architecture, which is potentially scalable and maintainable and may provide a useful framework for building more reliable and sustainably evolving research automation platforms.

Although the current version still has limitations, such as restricted access to paywalled literature, the need for expert‐verified chart data extraction, limited validation across research domains, and the requirement for expert interpretation of heterogeneity and subgroup findings, it suggests the potential of cutting‐edge AI technology to support evidence‐based research. By reducing repetitive workload in the evaluated workflow and allowing researchers to focus more on data quality control, scientific interpretation, and methodological judgment, this study provides a practical step toward an efficient, credible, and human‐verifiable paradigm for AI‐assisted evidence synthesis.

## Author Contributions

Yilin Yuan, Pingping Li, and Yang Wang contributed equally to this work. Yilin Yuan conceived and designed the study, developed the MacAma framework, Metaflow Analyzer, and all agent workflows, implemented the core software system, conducted the formal analysis and investigation, and drafted the original manuscript. Pingping Li contributed to data annotation, prompt engineering optimization, and experimental validation. Yang Wang contributed to study design, methodological guidance, and supervision of the research process. Boyuan Zheng, Yingshuang Liu, and Dongjin Yang contributed to algorithm optimization, data curation, and validation. Hai Lin, Min Wu, Qi Zhao, Jianwei Shuai, and Gen Yang supervised the project, acquired funding, and critically revised the manuscript. All authors have read and approved the final manuscript.

## Ethics Statement

The authors have nothing to report.

## Consent

The authors have nothing to report.

## Conflicts of Interest

The authors declare no conflicts of interest.

## Supporting information


Supporting Information S1

## Data Availability

The data that support the findings of this study are openly available in MacAma at https://github.com/YilinYuan/MacAma.
