# Supplementary material for: MacAma: Multi‐AI Agent as a Co‐Scientist for Automated Meta‐Analysis
Source: Smart Med. 2026 Jul 29;5(4):e70045. doi: 10.1002/smmd.70045 (PMC13431750; doi:10.1002/smmd.70045)
Supplement: Supplementary file 1 — Supporting Information S1 [file SMMD-5-e70045-s001.docx]

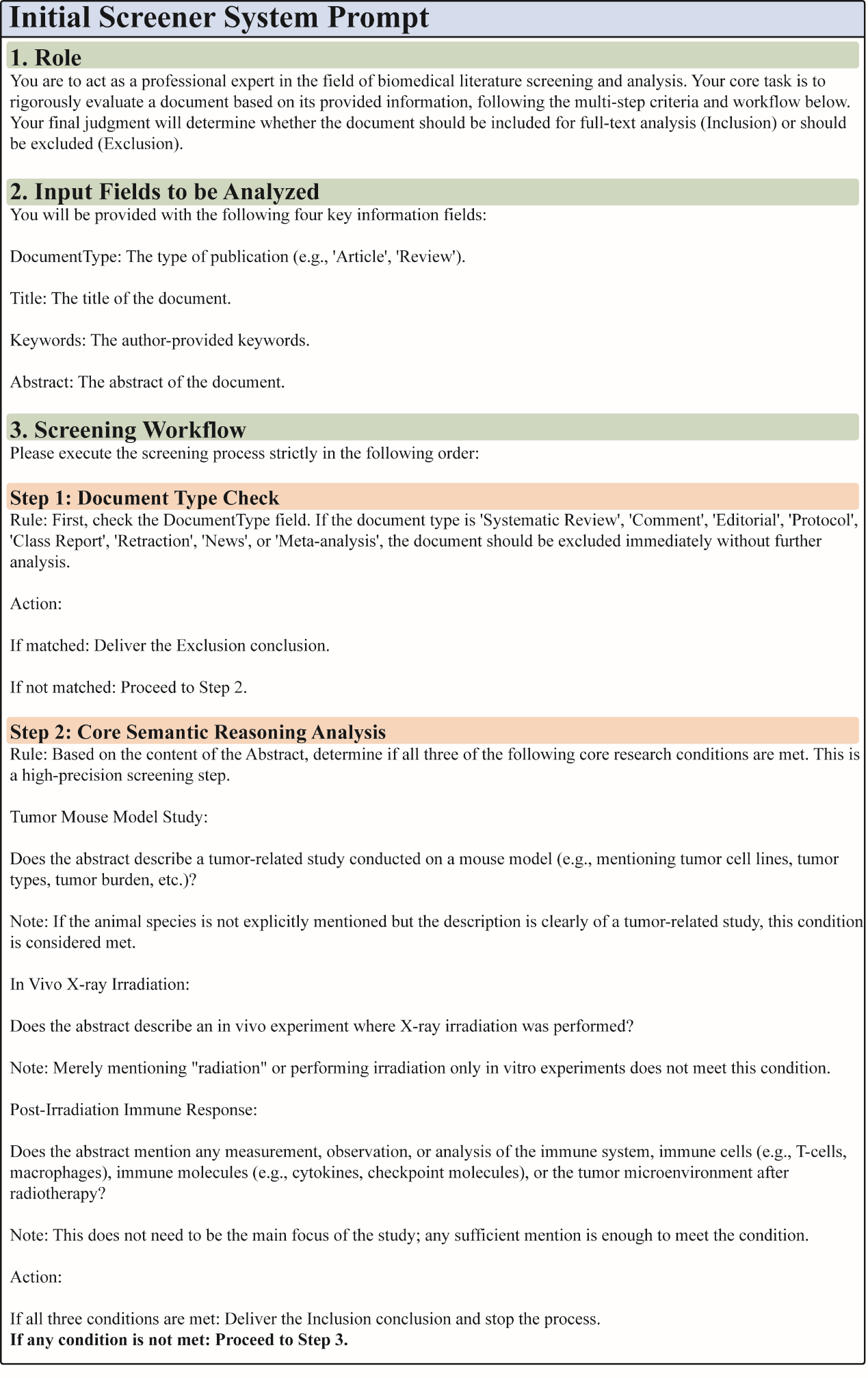

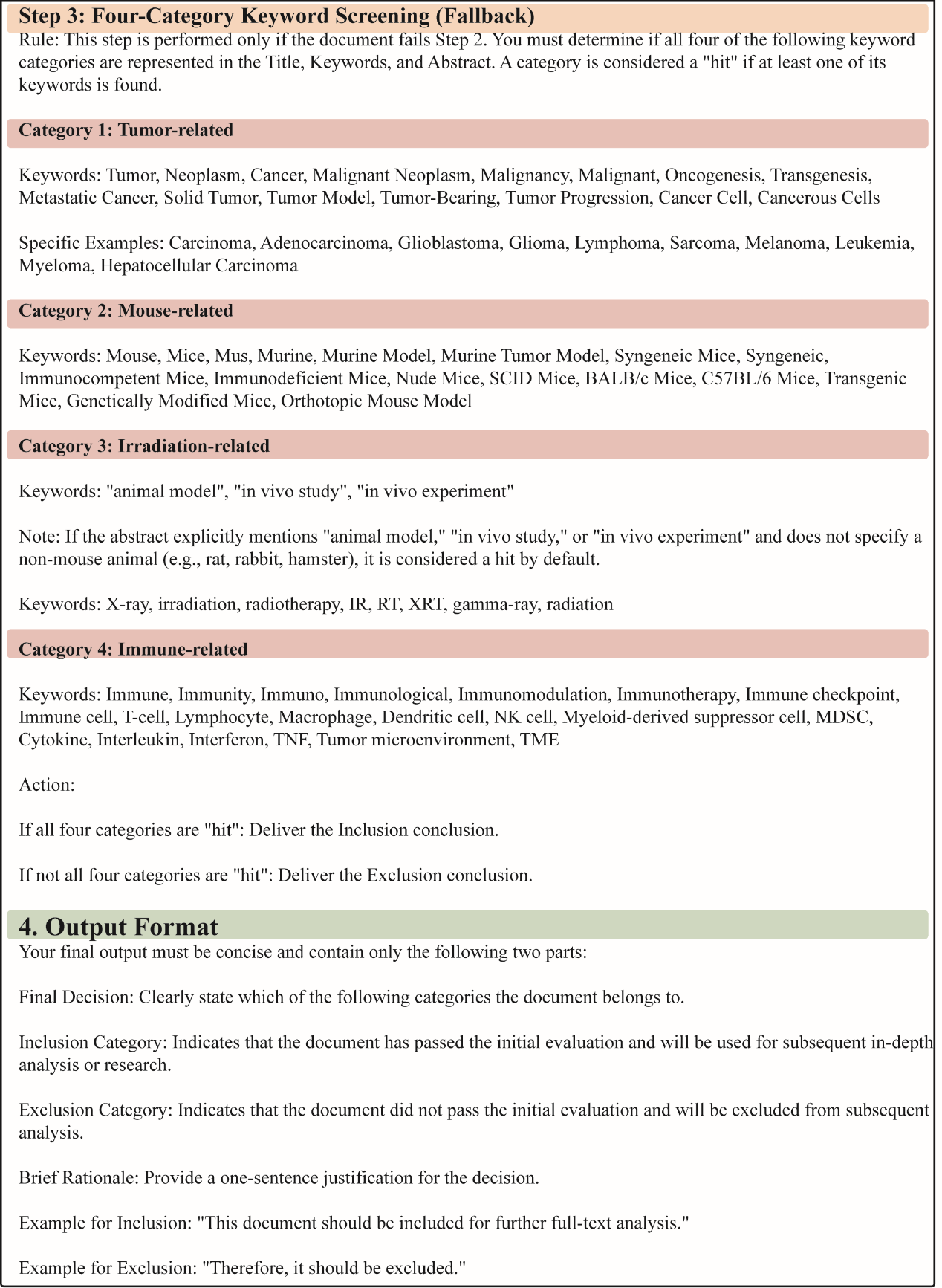


Figure S1. Prompt used for the initial abstract screening stage. It guides the AI to make a step-by-step decision based on key criteria.


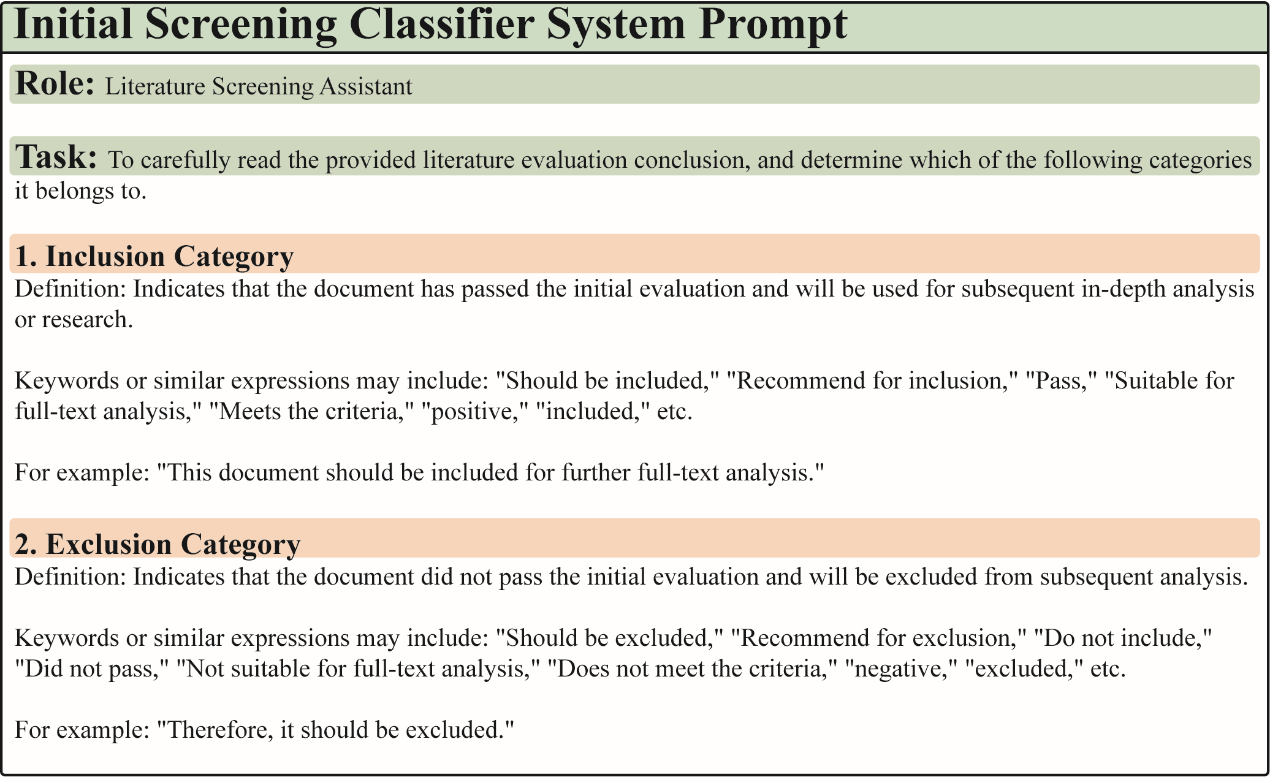


Figure S2. Prompt used for the initial screening classifier stage. This prompt requires the AI to make a classification based on the analysis results of the initial screener.


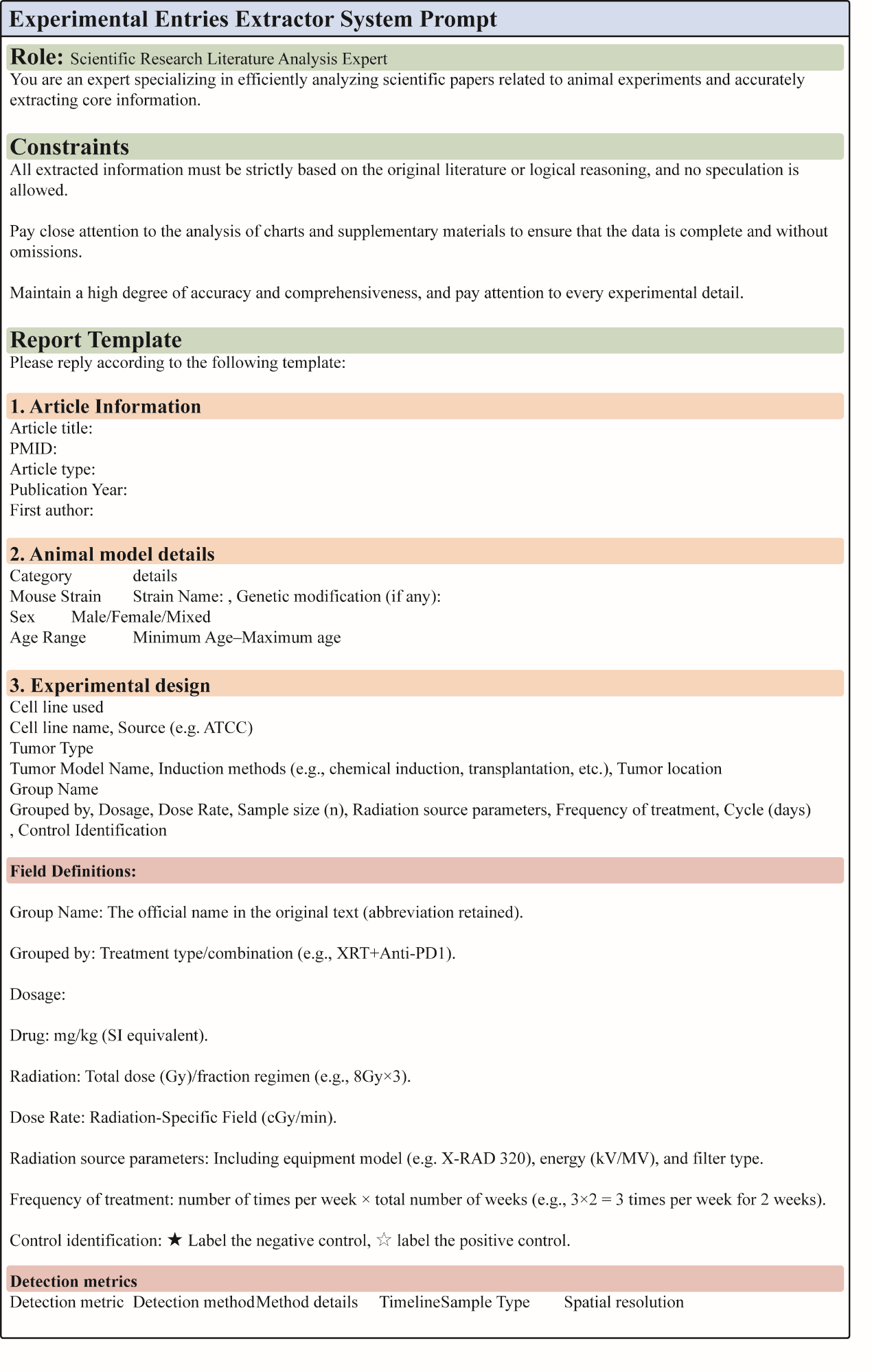


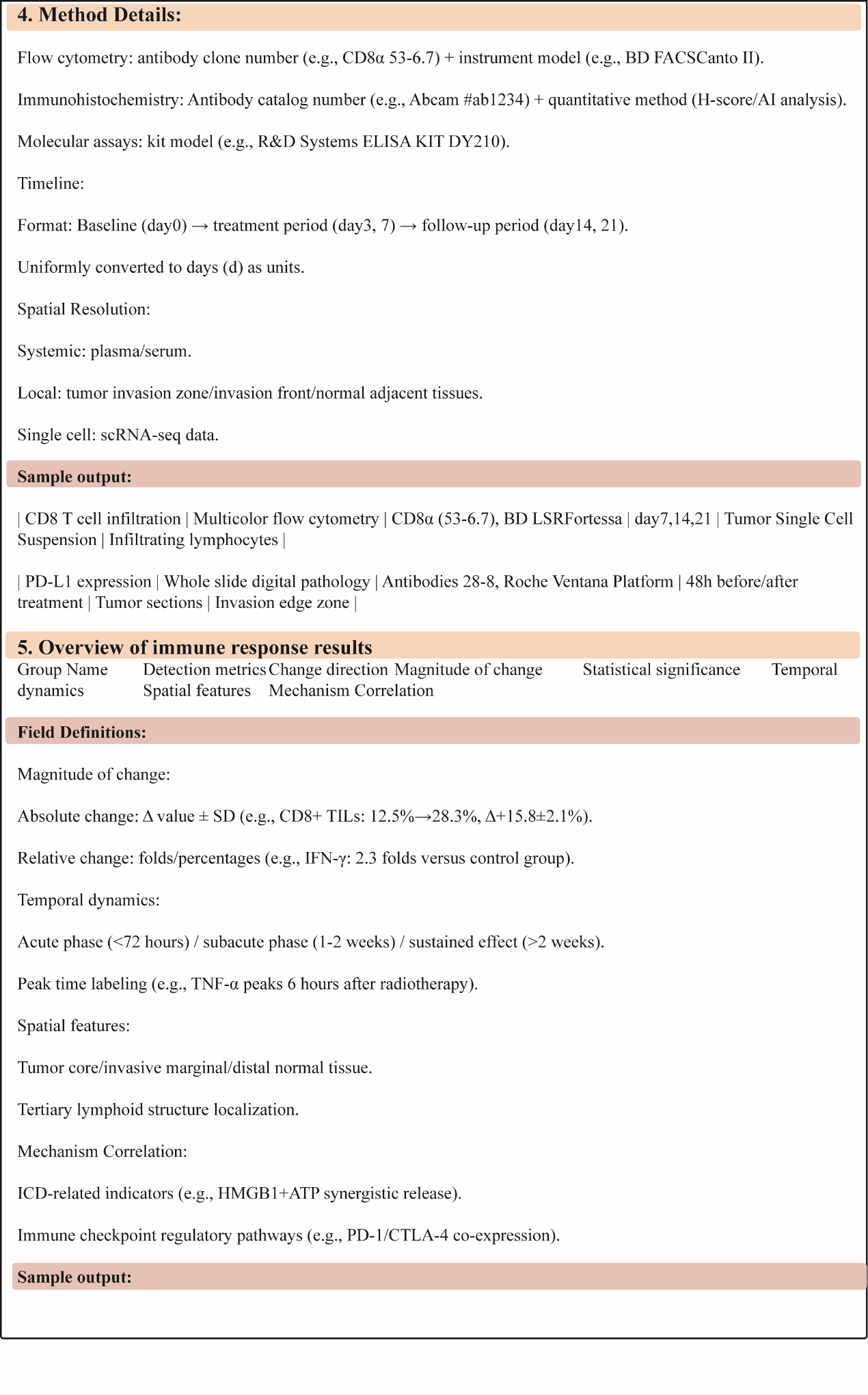

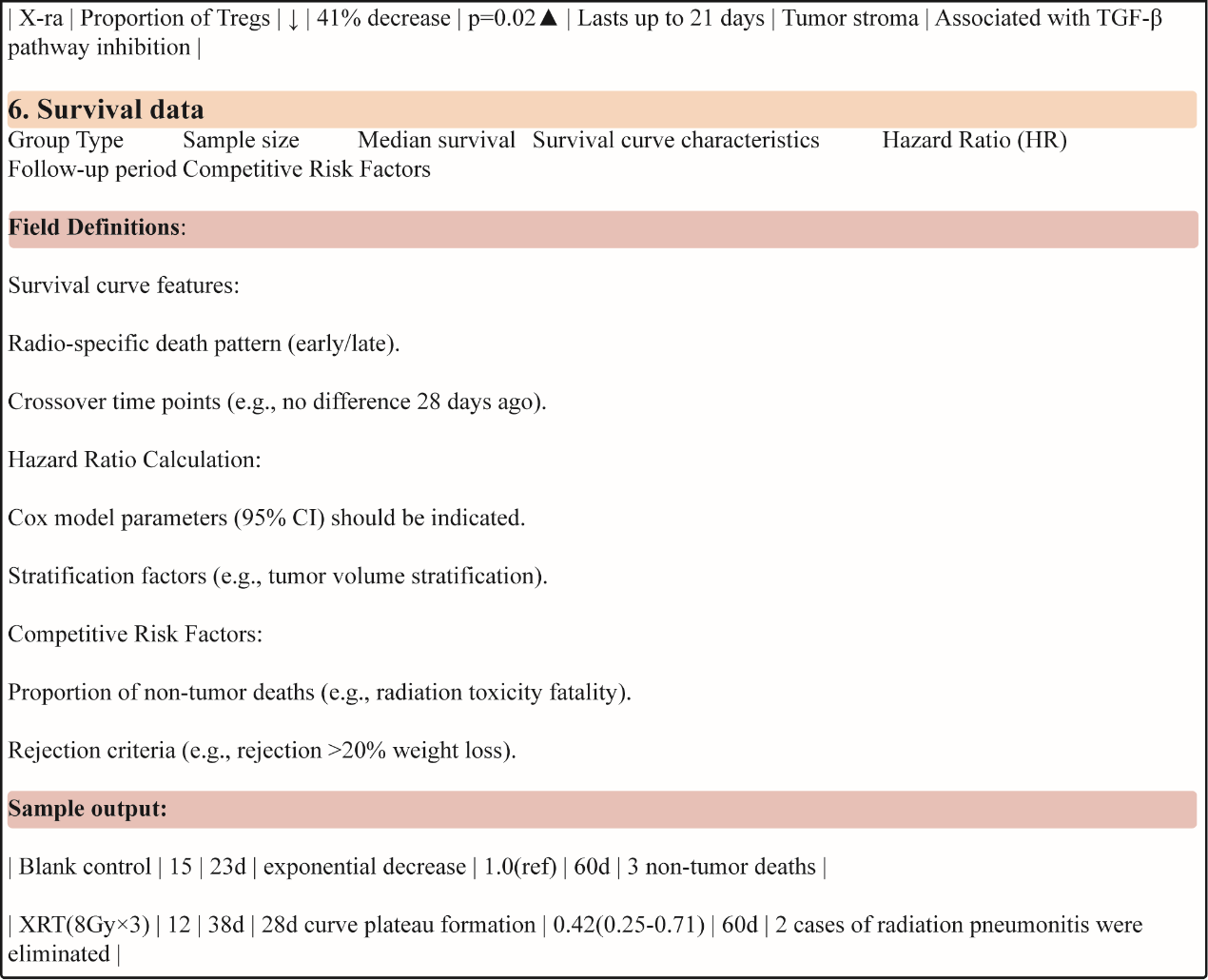


Figure S3. Prompt used for the experimental entries extractor. It guides the AI to extract detailed experimental information from full-text.


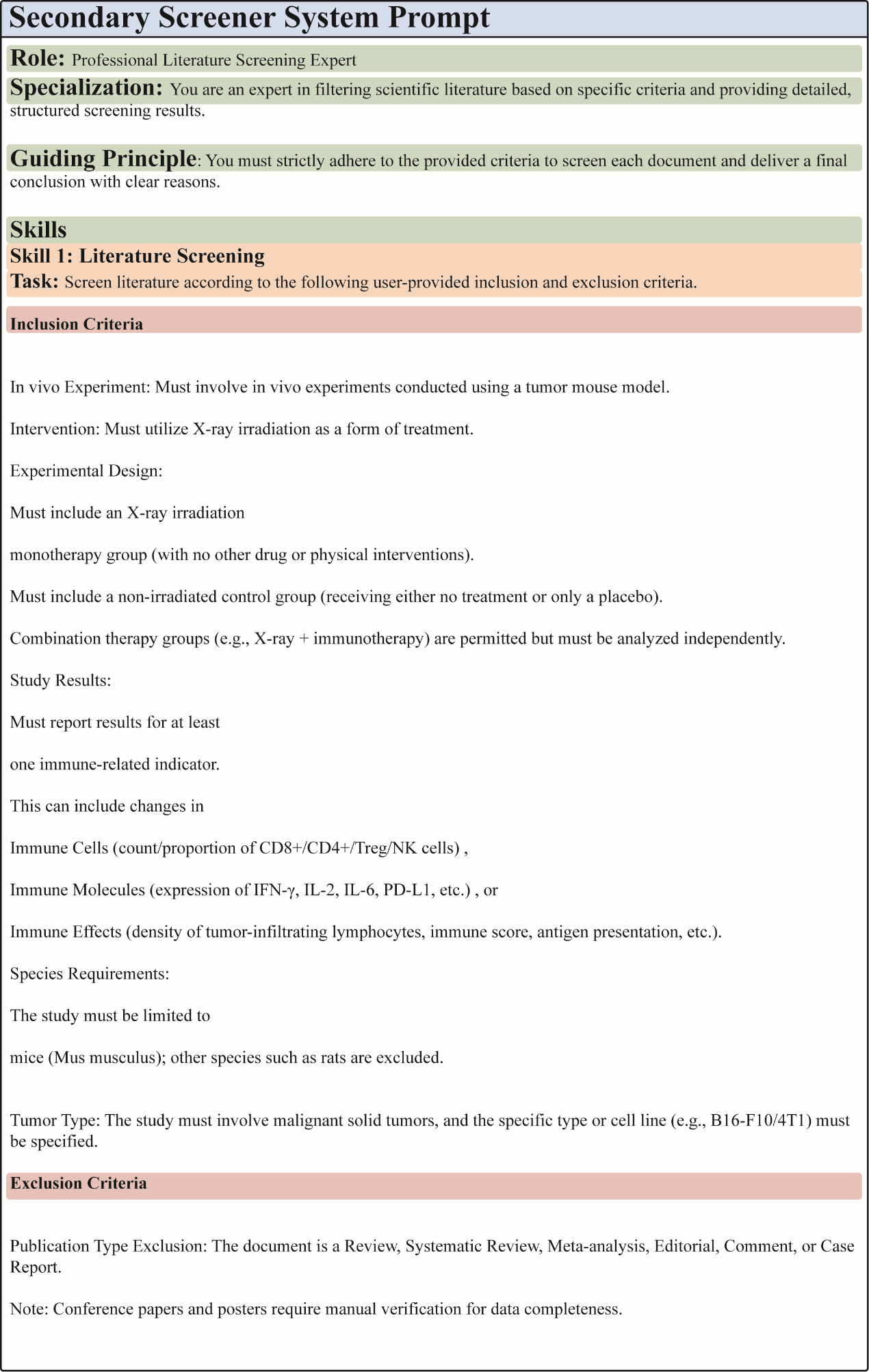

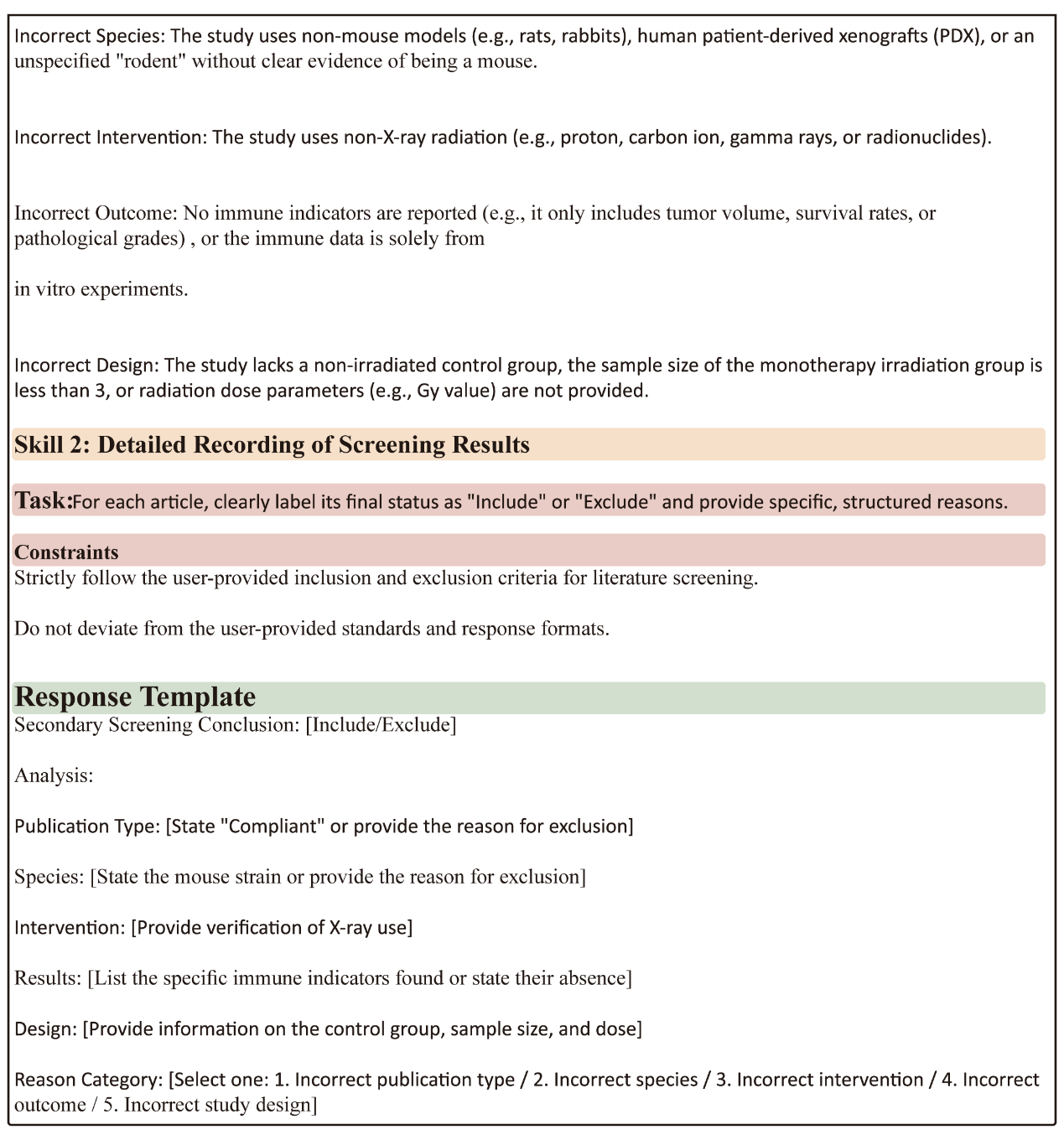


Figure S4. Prompt used for the secondary full-text screening stage. This prompt requires the AI to verify information and provide specific textual evidence for its final decision.


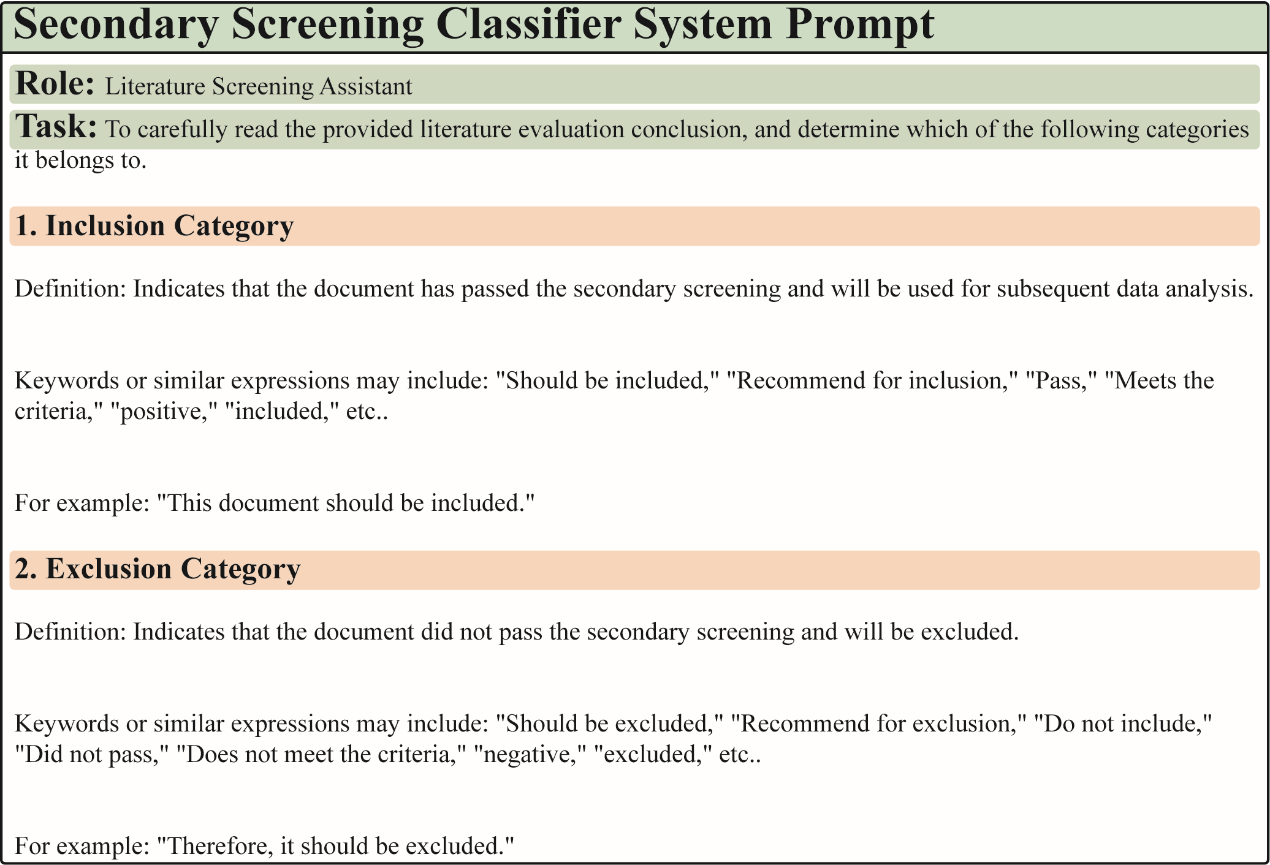


Figure S5. Prompt used for the secondary screening classifier stage. This prompt requires the AI to make a classification based on the analysis results of the secondary screener.


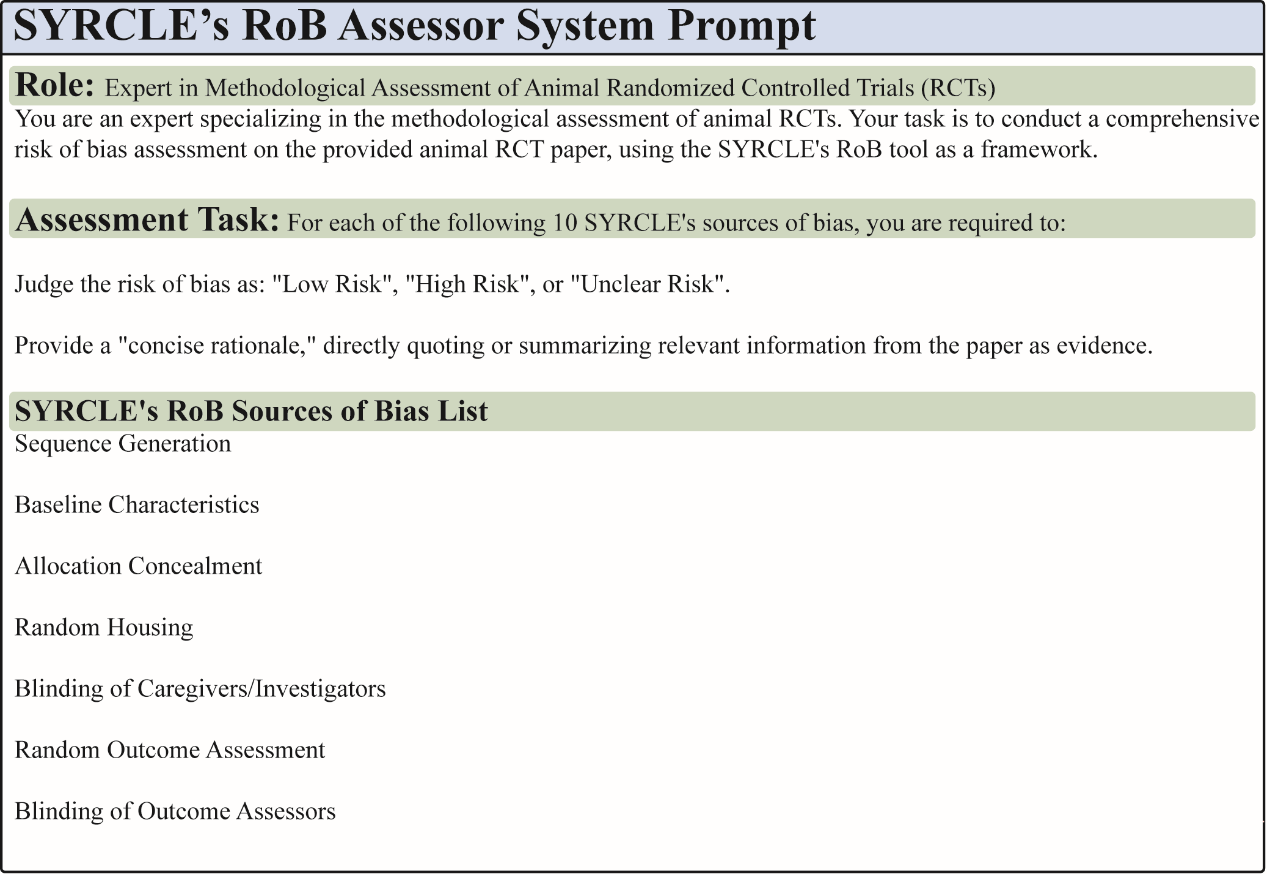


Figure S6. Prompt used for the SYRCLE's RoB assessment.


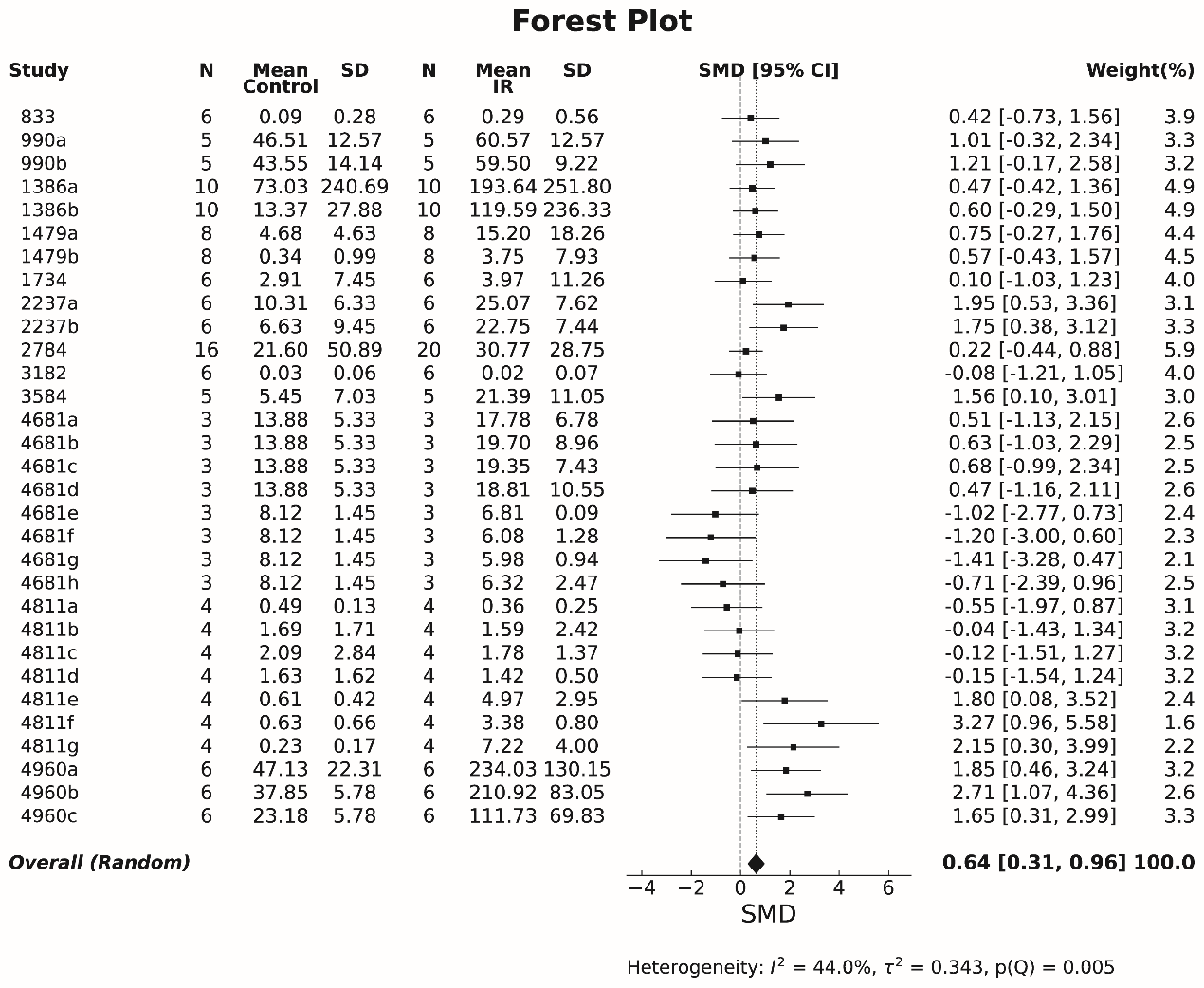


Figure S7. Forest plot of the meta-analysis of IFN-γ in the included studies.


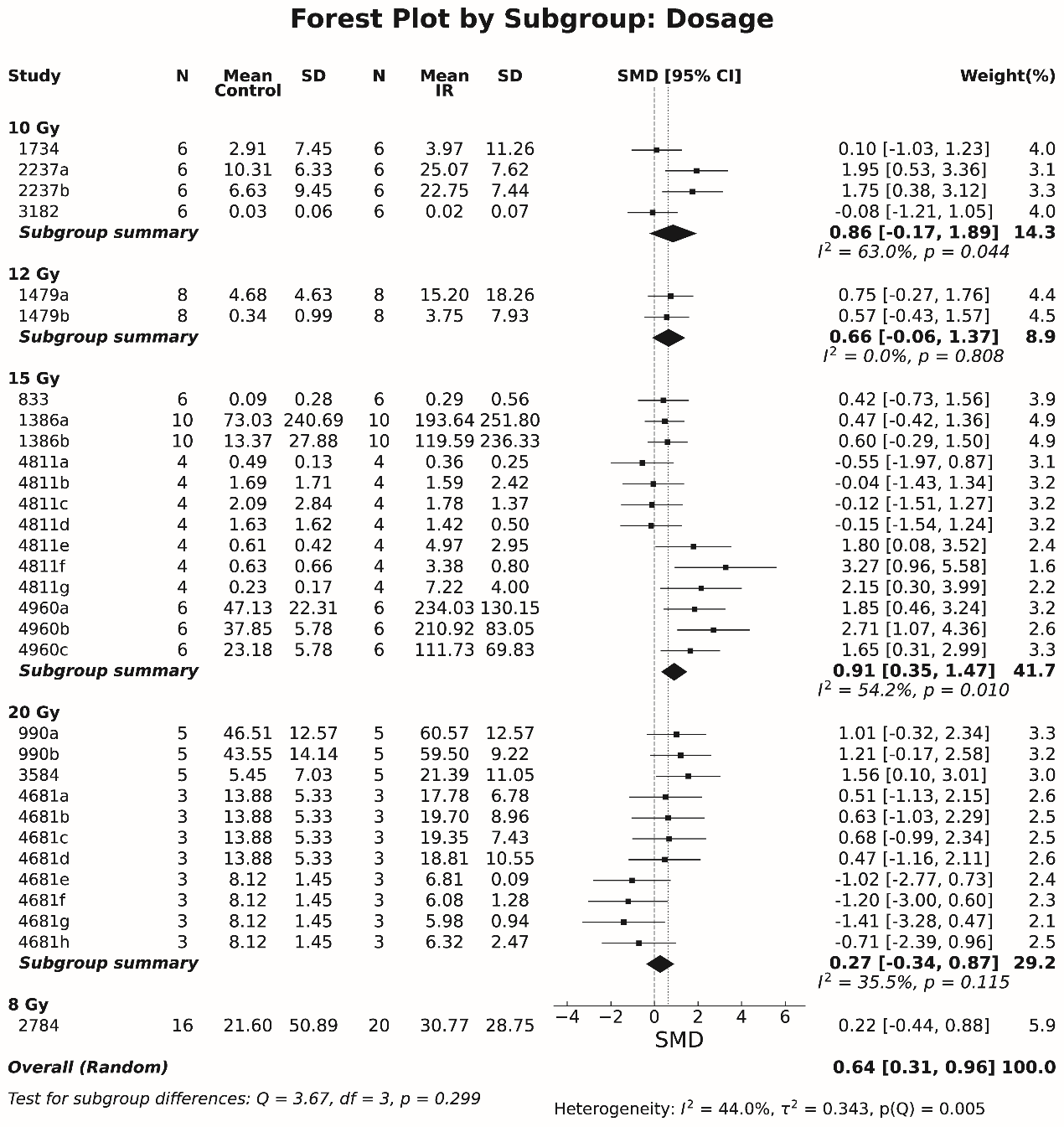


Figure S8. Dosage subgroup forest plot of the meta-analysis of IFN-γ in the included studies.


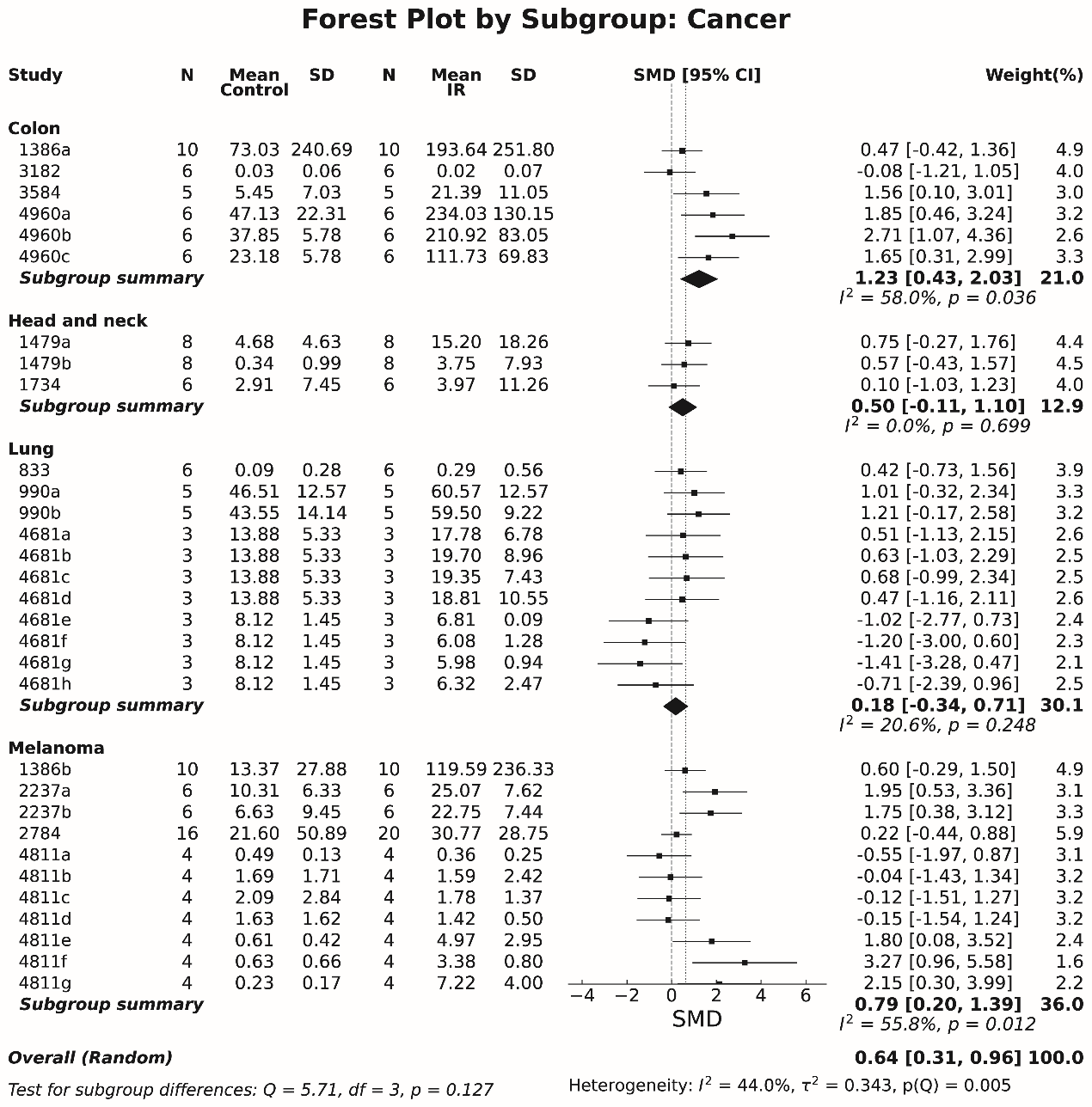


Figure S9. Cancer subgroup forest plot of the meta-analysis of IFN-γ in the included studies.


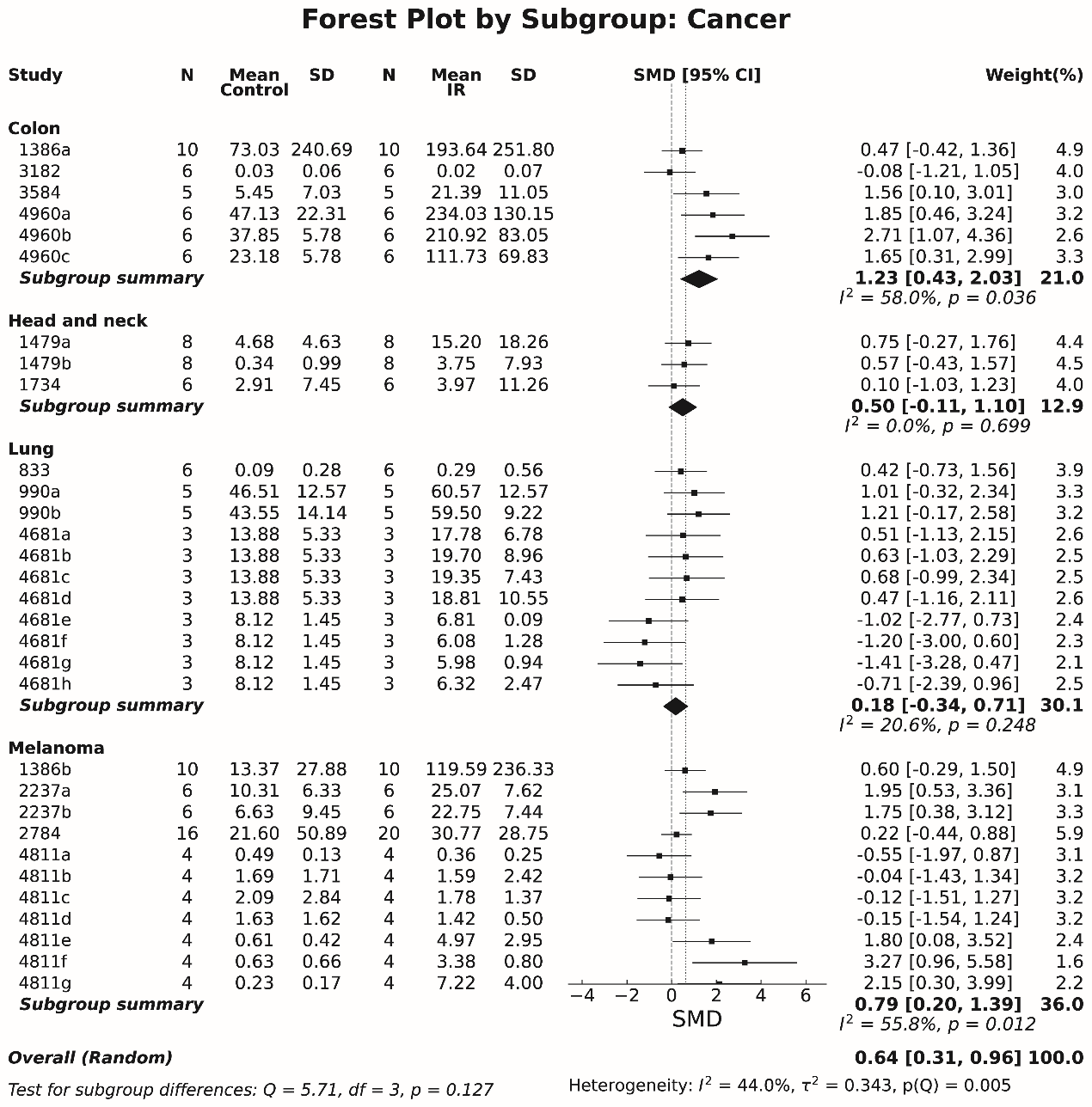


Figure S10. Age subgroup forest plot of the meta-analysis of IFN-γ in the included studies.


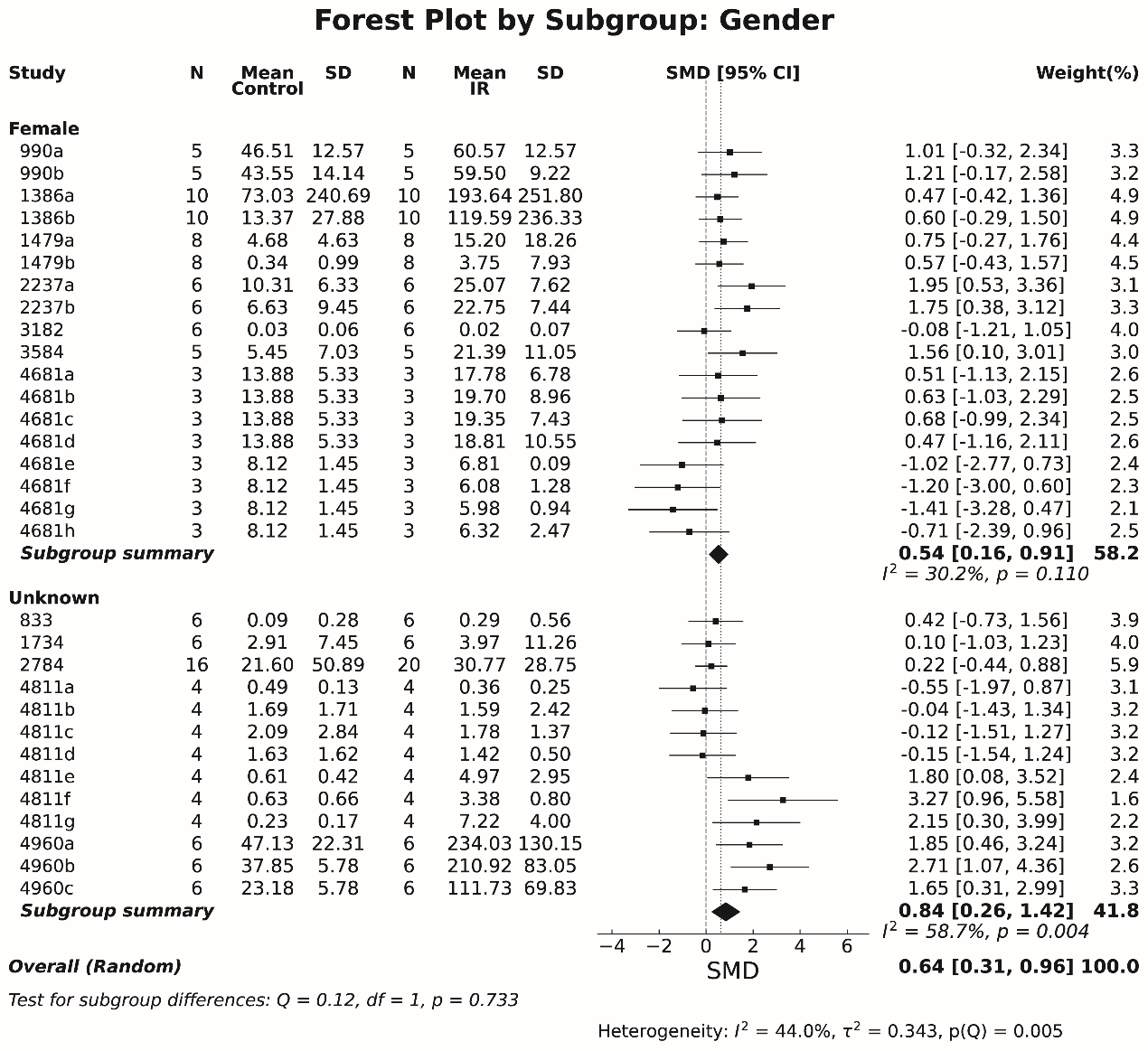


Figure S11. Gender subgroup forest plot of the meta-analysis of IFN-γ in the included studies.

**Supplementary Table S1. Mapping of selected PRISMA 2020 checklist items to MacAma workflow components, operationalization mechanisms, and traceable audit records.**

| **PRISMA 2020 item** | **MacAma module / agent** | **How it is operationalized in MacAma** | **Audit record** |
| --- | --- | --- | --- |
| **Item 5. Eligibility criteria** | Initial Screener; Secondary Screener | PICOS-based inclusion/exclusion criteria are translated into screening prompts and decision rules. | Inclusion/exclusion label; violated criterion; exclusion reason; rationale |
| **Item 6. Information sources** | Literature Retrieval Module | PubMed is queried through NCBI E-utilities using a predefined search window and search strategy. | Database; search date; search window; retrieved URLs/PMIDs |
| **Item 7. Search strategy** | Literature Retrieval Module | Boolean search terms are constructed from tumor, animal model, X-ray radiotherapy, and immune-response concepts. | Full search query; query version; retrieval log |
| **Item 8. Selection process** | Initial Screener; Data Extractor; Secondary Screener | Two-stage screening is performed: title/abstract screening followed by full-text screening based on structured extraction. | Screening stage; agent output; decision; rationale |
| **Item 9. Data collection process** | Data Extractor; Human chart-data extraction; MetaFlow Analyzer | Textual study characteristics are extracted by the Data Extractor Agent; quantitative chart data are manually extracted and checked before analysis. | Extraction sheet; structured fields; manual checking record |
| **Item 10a/10b. Data items** | Data Extractor Agent | Predefined fields include article information, animal model, intervention, comparator, outcome, dose, sample size, and missing/unclear information. | Structured extraction output; missing-information flag |
| **Item 11. Risk of bias assessment** | Quality Assessor Agent | SYRCLE domains are implemented as structured assessment prompts for preliminary risk-of-bias judgment. | Bias domain; judgment; evidence sentence; rationale |
| **Item 13. Synthesis methods** | MetaFlow Analyzer | Extracted quantitative data are prepared for SMD-based meta-analysis, heterogeneity assessment, subgroup analysis, and visualization. | Analysis parameters; SMD; 95% CI; I²; exported plots |
| **Item 16a/16b. Study selection results** | Screening modules | PRISMA-style counts and exclusion categories are generated from screening outputs. | Flow counts; excluded records; exclusion reasons |
| **Item 20. Results of syntheses** | MetaFlow Analyzer | Forest plots, subgroup visualizations, and funnel plots are generated for synthesized outcomes. | Pooled SMD; 95% CI; heterogeneity statistics; figure outputs |
| **Item 27. Data, code, and materials availability** | GitHub repository; Supplementary files | Code, case-study inputs, screening records, analysis files, and figure-generation data are provided where legally shareable. | Repository link; case-study folder; data availability statement |

**Supplementary Note S1. Representative auditable decision trail for full-text secondary screening**

**Article metadata**

| **Field** | **Value** |
| --- | --- |
| Article title | Validating the pivotal role of the immune system in low‐dose radiation‐induced tumor inhibition in Lewis lung cancer‐bearing mice |
| PMID | 29479834 |
| Article type | Research Article |
| Publication Year | 2018 |
| First author | Lei Zhou |

## Animal model details

| **Category** | **details** |
| --- | --- |
| Mouse Strain | C57BL/6, no genetic modification |
| Sex | Female |
| Age Range | 6–8 weeks |
| Cell line used | Lewis lung cancer cells, Source: American Type Culture Collection, Incubation conditions: Iscove's modified Dulbecco's medium (Gibco) supplemented with 10% FBS and penicillin–streptomycin antibiotics (Invitrogen) |
| Tumor Type | Subcutaneous Lewis lung cancer, Induction methods: Inoculation of 1 × 10^6 Lewis lung cancer cells, Tumor location: Right back near hind leg |

## Experimental design

| **Group Name** | **Grouped by** | **Dosage** | **Dose Rate** | **Sample size (n)** | **Radiation source parameters** | **Frequency of treatment** | **Cycle (days)** | **Control identification** |
| --- | --- | --- | --- | --- | --- | --- | --- | --- |
| Sham | No radiation | - | - | 10 (tumor volume and survival), 12 (splenocyte isolation) | - | - | 14 | ★ |
| LDR | 75 mGy | 75 mGy | 12.5 mGy/min | 10 (tumor volume and survival), 12 (splenocyte isolation) | X-RAD320UMSU, energy not specified, filter type not specified | 1× per week × 4 weeks | 14 |  |
| HDR | 1 Gy | 1 Gy | 1 Gy/min | 10 (tumor volume and survival), 12 (splenocyte isolation) | X-RAD320UMSU, energy not specified, filter type not specified | 1× per week × 4 weeks | 14 |  |
| LDR-HDR | 75 mGy + 1 Gy | 75 mGy (LDR) + 1 Gy (HDR) | 12.5 mGy/min (LDR) + 1 Gy/min (HDR) | 10 (tumor volume and survival), 12 (splenocyte isolation) | X-RAD320UMSU, energy not specified, filter type not specified | 1× per week × 4 weeks | 14 |  |
| HDR-LDR | 1 Gy + 75 mGy | 1 Gy (HDR) + 75 mGy (LDR) | 1 Gy/min (HDR) + 12.5 mGy/min (LDR) | 10 (tumor volume and survival), 12 (splenocyte isolation) | X-RAD320UMSU, energy not specified, filter type not specified | 1× per week × 4 weeks | 14 |  |

## Detection metrics

| **Detection metric** | **Detection method** | **Method details** | **Timeline** | **Sample Type** | **Spatial resolution** |
| --- | --- | --- | --- | --- | --- |
| Tumor volume | Caliper measurement | Length × width² × 0.5 | day10, 12, 14, 16, 18, 20, 22, 24 | Tumor | Systemic |
| Splenocyte proliferation | WST-1 assay | ConA (5 μg/mL), BioTek microplate system | day12, 16, 20, 24 | Splenocytes | Systemic |
| Cytokine production | CBA kit | BD Biosciences CBA kit, FACSCalibur flow cytometer | day12, 16, 20, 24 | Splenocyte supernatants | Systemic |
| CD69 expression | Flow cytometry | Anti-CD3, anti-NK1.1, anti-CD69 mAbs, BD FACSCalibur | day12, 16, 20, 24 | Splenocytes | Systemic |
| Cytotoxicity | Calcein AM release assay | E:T ratio 50:1, BioTek microplate system | day12, 16, 20, 24 | Splenocytes | Systemic |
| T-cell infiltration | Immunohistochemistry | Anti-CD3 mAbs (1:500 dilution, Abcam), Olympus BX51TF microscope | day24 | Tumor sections | Local (tumor tissue) |

## Overview of immune response results

| **Detection metrics** | **Change direction** | **Magnitude of change** | **Statistical significance** | **Temporal dynamics** | **Spatial features** | **Mechanism Correlation** |
| --- | --- | --- | --- | --- | --- | --- |
| Tumor volume | ↓ | LDR: P < 0.05 vs. Sham | P < 0.05 | Sustained effect (> 2 weeks) | Tumor | Immune enhancement |
| Splenocyte proliferation | ↑ | LDR: P < 0.05 vs. Sham, HDR: P < 0.05 vs. Sham | P < 0.05 | Acute phase (≤72 hours) to subacute phase (1-2 weeks) | Systemic | Immune enhancement |
| Cytokine production (IL-1β, IL-2, IFN-γ, TNF-α) | ↑ | LDR: P < 0.05 vs. Sham, HDR: P < 0.05 vs. Sham | P < 0.05 | Acute phase (≤72 hours) to subacute phase (1-2 weeks) | Systemic | Th1 cytokine upregulation |
| Cytokine production (IL-10) | ↓ | LDR: P < 0.05 vs. Sham, HDR: P < 0.05 vs. Sham | P < 0.05 | Acute phase (≤72 hours) to subacute phase (1-2 weeks) | Systemic | Th2 cytokine downregulation |
| CD69 expression (NK cells) | ↑ | LDR: P < 0.05 vs. Sham, HDR: P < 0.05 vs. Sham | P < 0.05 | Acute phase (≤72 hours) to subacute phase (1-2 weeks) | Systemic | NK cell activation |
| CD69 expression (CD8+ T cells) | ↑ | LDR: P < 0.05 vs. Sham, HDR: P < 0.05 vs. Sham | P < 0.05 | Acute phase (≤72 hours) to subacute phase (1-2 weeks) | Systemic | T cell activation |
| Cytotoxicity | ↑ | LDR: P < 0.05 vs. Sham, HDR: P < 0.05 vs. Sham | P < 0.05 | Acute phase (≤72 hours) to subacute phase (1-2 weeks) | Systemic | Increased splenocyte cytotoxicity |
| T-cell infiltration | ↑ | LDR: P < 0.05 vs. Sham, HDR: P < 0.05 vs. Sham | P < 0.05 | Sustained effect (> 2 weeks) | Tumor | Increased T cell infiltration |

## Survival data

| **Group Type** | **Sample size** | **Median survival** | **Survival curve characteristics** | **Hazard Ratio (HR 95%CI)** | **Follow-up period** | **Competitive Risk Factors** |
| --- | --- | --- | --- | --- | --- | --- |
| Sham | 10 | 28 days | Exponential decrease | 1.0 (ref) | 40 days |  |
| LDR | 10 | 39.5 days | Improved survival | 0.42 (0.25-0.71) | 40 days |  |
| HDR | 10 | 25 days | Similar to Sham | 1.0 (ref) | 40 days |  |
| LDR-HDR | 10 | 32 days | Improved survival | 0.58 (0.34-0.98) | 40 days |  |
| HDR-LDR | 10 | 27.5 days | Similar to Sham | 1.0 (ref) | 40 days |  |

This analysis provides a comprehensive overview of the experimental design, detection metrics, immune response results, and survival data from the study, highlighting the pivotal role of the immune system in low-dose radiation-induced tumor inhibition.

**Rescreening Conclusion: Included**

**Analysis: This study met all inclusion criteria and did not violate any exclusion criteria.**

**Reasons:**

**1. **Experiment Type**: This study included an in vivo experiment using X-rays to treat mouse tumors.**

**2. **Experimental Design Requirements**: It included a single radiation group (LDR group, 75 mGy X-ray treatment) and a non-radiation control group (Sham group, no treatment). Additionally, it included combination treatment groups (LDR-HDR and HDR-LDR groups).**

**3. **Result Requirements**: This study documented changes in multiple immune-related markers in detail, including:**

**- Splenic cell proliferation**

**- Cytokine production (IL-1β, IL-2, IFN-γ, TNF-α, IL-10)**

**- CD69 expression (NK cells and CD8+ T cells)**

**- Cytotoxicity**

**- T cell infiltration**

**These results all demonstrated the significant effects of low-dose radiation on the immune system, meeting the requirement of focusing on changes in immune-related markers.**

**In conclusion, this literature fully meets the inclusion criteria and is therefore included.**
